# Live Attenuated *Salmonella enterica* Expressing and Releasing Cell-Permeable Bax BH3 Peptide Through the MisL Autotransporter System Elicits Antitumor Activity in a Murine Xenograft Model of Human B Non-hodgkin's Lymphoma

**DOI:** 10.3389/fimmu.2019.02562

**Published:** 2019-11-14

**Authors:** Armando Alfredo Mateos-Chávez, Paola Muñoz-López, Elayne Irene Becerra-Báez, Luis Fernando Flores-Martínez, Diego Prada-Gracia, Liliana Marisol Moreno-Vargas, Guillermina Juliana Baay-Guzmán, Uriel Juárez-Hernández, Bibiana Chávez-Munguía, Lourdes Cabrera-Muñóz, Rosendo Luria-Pérez

**Affiliations:** ^1^Unit of Investigative Research on Oncological Diseases, Children's Hospital of Mexico Federico Gomez, Mexico City, Mexico; ^2^Posgrado en Biomedicina y Biotecnología Molecular, Escuela Nacional de Ciencias Biológicas, Instituto Politécnico Nacional, Mexico City, Mexico; ^3^Research Unit on Computational Biology and Drug Design, Children's Hospital of Mexico Federico Gomez, Mexico City, Mexico; ^4^Department of Molecular Biomedicine, Center for Research and Advanced Studies of the National Polytechnic Institute, Mexico City, Mexico; ^5^Department of Infectomics and Molecular Pathogenesis, Center for Research and Advanced Studies of the National Polytechnic Institute, Mexico City, Mexico; ^6^Department of Clinical and Experimental Pathology, Children's Hospital of Mexico Federico Gomez, Mexico City, Mexico

**Keywords:** non-Hodgkin's lymphoma, *Salmonella enterica*, cancer, Bax BH3 peptide, apoptosis, immunotherapy, MisL autotransporter

## Abstract

The survival of patients with non-Hodgkin's lymphoma (NHL) has substantially improved with current treatments. Nevertheless, the appearance of drug-resistant cancer cells leads to patient relapse. It is therefore necessary to find new antitumor therapies that can completely eradicate transformed cells. Chemotherapy-resistant cancer cells are characterized by the overexpression of members of the anti-apoptotic B-cell lymphoma 2 (Bcl-2) protein family, such as Bcl-_XL_, Bcl-2, and Mcl-1. We have recently shown that peptides derived from the BH3 domain of the pro-apoptotic Bax protein may antagonize the anti-apoptotic activity of the Bcl-2 family proteins, restore apoptosis, and induce chemosensitization of tumor cells. In this study, we investigated the feasibility of releasing this peptide into the tumor microenvironment using live attenuated *Salmonella enterica*, which has proven to be an ally in cancer therapy due to its high affinity for tumor tissue, its ability to activate the innate and adaptive antitumor immune responses, and its potential use as a delivery system of heterologous molecules. Thus, we expressed and released the cell-permeable Bax BH3 peptide from the surface of *Salmonella enterica* serovar Typhimurium SL3261 through the MisL autotransporter system. We demonstrated that this recombinant bacterium significantly decreased the viability and increased the apoptosis of Ramos cells, a human B NHL cell line. Indeed, the intravenous administration of this recombinant *Salmonella enterica* elicited antitumor activity and extended survival in a xenograft NHL murine model. This antitumor activity was mediated by apoptosis and an inflammatory response. Our approach may represent an eventual alternative to treat relapsing or refractory NHL.

## Introduction

Cancer is a worldwide primary health problem, with an annual mortality rate of over 8 million individuals (1). Within the broad malignancy spectrum, non-Hodgkin's lymphoma (NHL) represents 90% of all lymphomas and is one of the most frequent neoplasias in the world (1, 2). NHL arises from the malignant transformation of immature and mature immune cells, compromising B lymphocytes in 86% of cases, and T and NK lymphocytes in 14% (3). Fortunately, chemotherapy, radiotherapy, and immunotherapy have increased patient overall survival to over 80% at 5 years (4, 5). However, complete treatment success is limited by the development of drug resistance, a situation with a dire prognosis and with limited possibilities of a cure (6). It is therefore necessary to find new antitumor therapies that can completely eradicate drug-resistant transformed cells (7).

One of the mechanisms of drug resistance in cancer is the modification of the genes and proteins controlling the mitochondrial pathway of apoptosis, such as members of the B-cell lymphoma 2 (Bcl-2) family (8, 9). This family includes pro-apoptotic BH3-only proteins (Bid, Bim, Puma, Noxa, Bad, Bmf, Hrk, and Bik), anti-apoptotic proteins (Bcl-2, Bcl-_XL_, Bcl-w, Mcl-1, and A1), and effector pro-apoptotic proteins (Bax, Bak, and Bok). Overexpression of anti-apoptotic proteins such as Bcl-2, Bcl-_XL_, and Mcl-1 has been associated with drug resistance in human tumor cell lines (10–12), including NHL cells (13–16). Structural analysis of proteins in the Bcl-2 family has shown an interaction between them via a hydrophobic groove formed by the BH domains (17–19), and the overexpression of anti-apoptotic proteins promotes binding to the pro-apoptotic effector proteins Bax or Bak and inhibits their polymerization on the mitochondrial membrane, thus precluding the release of cytochrome c and the initiation of apoptosis (20, 21). Interaction of these proteins has led to the proposal of eliminating tumor cells that are resistant to apoptosis by blocking the activity of anti-apoptotic proteins with peptides derived from the BH3 domain of pro-apoptotic proteins such as Bak, Bax, Noxa, and Bid that once bound to the Bcl-_XL_, Bcl-2, and Mcl-1 proteins, antagonizing their function (22–24). *In vitro* assays using hydrophobic peptides from the BH3 domain of the proteins Bax, Bad, and Bak, once coupled to the fusogenic peptide of the antennapedia protein (to make them permeable to head and neck squamous cell carcinoma tumor cells), antagonized the Bcl-_XL_ and Bcl-2 activity and restored the apoptosis (25). Furthermore, the small molecules that mimic the function of the BH3-only proteins have been tested in clinical trials, and even the inhibitor of Bcl-2 activity, Venotoclax/ABT-199, was recently approved by the U.S. Food and Drug Administration (FDA) for the treatment of chronic lymphocytic leukemia (CLL) (26, 27).

In spite of their efficacy and promising results, BH3 domain peptides and the molecules mimicking the BH3 domain still need to be specifically and selectively directed toward the tumor microenvironment in order to decrease side effects. Several strategies have been attempted to overcome this problem, so in this study, we have suggested the use of a live attenuated bacterial vector, *Salmonella enterica* serovar Typhimurium strain SL3261, which has been proven to be an ally in the therapy of cancer due to its high affinity for tumor tissue (28, 29), its ability to activate the innate and adaptive antitumor immune responses (30), and its potential use as a delivery system, since once in the tumor microenvironment, it becomes a true factory of heterologous molecules (31, 32). We recently demonstrated the ability of *Salmonella enterica* to carry and transfer plasmids into tumor cells (bactofection). Transferred plasmid encoding a peptide from the BH3 domain of the pro-apoptotic Bax protein antagonized the anti-apoptotic activity of the Bcl-2 family proteins, restored apoptosis, and induced chemosensitization of tumor cells (33). In this study, we evaluated the feasibility for the cell-permeable Bax BH3 peptide [Tag peptide (T) bound to Bax BH3 peptide (X) and the fusogenic peptide (P)] expressed and released from the surface of *Salmonella enterica* serovar Typhimurium strain SL3261 through the MisL autotransporter system (34) (*Salmonella enterica* L-STXP) to promote apoptosis signaling and the death of NHL tumor cells. Our results demonstrated that *Salmonella enterica* L-STXP significantly decreased the viability and increased apoptosis in Ramos cells, a human B NHL cell line. Indeed, the intravenous administration of this recombinant bacterium elicited antitumor activity and extended survival in a murine xenograft model of human B NHL. This antitumor activity was mediated by apoptosis and an inflammatory response. Taken together, our results suggest that the live attenuated *Salmonella enterica* serovar Typhimurium strain SL3261 expressing and releasing cell-permeable Bax BH3 peptide through the MisL autotransporter system may represent an eventual alternative to treat relapsing or refractory NHL.

## Materials and Methods

### Molecular Modeling by Homology

To generate the model of the L-SXTP chimera [MisL autotransporter system = L (35) (NCBI Reference Sequence NP_462656.1), OmpT cleavage recognition site = S (34), Bax BH3 peptide = X (25), Flag peptide = T (34), and fusogenic peptide = P (34, 36)], we used two independent strategies and then chose the consensus model. On the one hand, we used an assembly of large rigid fragments, including the entire folding, obtained from similar structures aligned by means of their primary and secondary sequences. This methodology cuts and pastes fragments of the peptide skeleton of known structures (SWISS-MODEL) (37, 38). On the other hand, we used modeling for the satisfaction of molecular constraints extracted from databases and similar structures aligned. This method helps produce a set of structures for the A sequence, all of them compatible with the restrictions observed in the templates (MODELER) (39, 40). All subunits (L, S, X, T, and P) were modeled separately using molecular modeling by homology. As templates, we used three-dimensional (3-D) structures from the PDB (http://www.rcsb.org/pdb). The MisL autotransporter system was modeled using a library of segments that contained structural information of the following coordinate files: 4MEE, 3KVN, 3SLJ, 3QQ2, 3AEH, 1UYN, 2QOM, 3ML3, 1DAB, 3H09—all of them with identities in sequence between 13 and 43%. The Bax BH3 peptide, coupled at OmpT peptide, was modeled using the 3-D structure of BCL-2 in complex with a Bax BH3 peptide (PDB code: 2XA0, 2.7 Å resolution) (41) and the Bax BH3-in-Groove dimer (PBD: 4BDU, 2.9 Å resolution) (42). The fusogenic peptide, coupled at Flag peptide, was modeled using the 3-D coordinates deposited in the following ID PDBs: 5FN4, 6IJO, and 5OA1.

#### Geometry Optimization of the Proposed Models

Once the 3-D models were prepared, hydrogen atoms were added, and side chain orientations were optimized through energy minimization using the steepest descent method, employing 2,000 cycles using the CHARMM27 parameters found in NAMD (43). Next, the complex was assembled and finally was embedded in a biological membrane using the set of CHARM-GUI programs (44, 45). The positioning in the membrane, in terms of inclination angle and hydrophobic thickness, was calculated using the Orientations of Proteins in Membranes (OPM) database (46). We also obtained information on the number of transmembrane secondary structure segments and their composition.

#### Stereochemical Quality Evaluation of the Models

Coordinate files of the 3-D models were sent to MolProbity (47) to produce a Ramachandran plot (ϕ and ψ angles), reflecting polypeptide chain distortion in the non-allowed region. We also sent the coordinate files to RAMPAGE to identify side chains with less common conformations possibly because of local protein tension (48). The quality of the models was further validated using two additional tools: ProQ3/ProQ3D (49, 50) and QMEAN (51).

### Bacterial Strains and Oligonucleotides

The following bacterial strains were used: *Escherichia coli* strain DH5-α (*E. coli* DH5-α) (Gibco, BRL, Gaithersburg, MD, USA) and *Salmonella enterica* serovar Typhimurium strain SL3261, a mutant at Aro A (*Salmonella enterica* SL3261) (34, 52). The employed oligonucleotide sequences used in this study are summarized in Table 1. The oligonucleotide sequences encoded the following peptides: fusogenic [P, EAAAAAEAAAAAEAAAAAEAAAAA (34, 36)], Flag [T, DYKDDDDK (34)], Bax BH3 [X, STKKLSECLKRIGDELDSNM (25)], OmpT protease cleavage site [S, KRPGGGGGKRGGGGGPKR (34)].

**Table 1:** Oligonucleotides used in this study.

**Name**	**Sequence**	**Characteristics**
FP 1	5′CTAGATGCGAAGCGGCCGCTGCAGCGGAAGCCGCAGCTGCGGCAGAA GCTGCGGCAGCCGCTGAAGCGGCTGCCGCGGCAGCTAGCGTCGACG3′	Forward. Encodes the synthetic fusogenic peptides and *Xba*I, *Nhe*I, *Sal*I, and *BamH*I restriction sites (underlined).
FP 2	5′GATCCGTCGACGCTAGCTGCCGCGGCAGCCGCTTCAGCGGCTGCCGCA GCTTCTGCCGCAGCTGCGGCTTCCGCTGCAGCGGCCGCTTCGCAT3′	Reverse. Encodes the synthetic fusogenic peptides and *Xba*I, *Nhe*I, *Sal*I, and *BamH*I restriction sites (underlined).
FLAG1	5′CTAGAGATTATAAAGATGACGATGACAAAGCTAGCATGCATG3′	Forward. Encodes the molecular Tag Flag and *Xba*I, *Nhe* I, *Nsi*I, and *BamH* I restriction sites (underlined).
FLAG2	5′GATCCATGCATGCTAGCTTTGTCATCGTCATCTTTATAATCT3′	Reverse. Encodes the molecular Tag Flag and *Xba*I, *Nhe*I, *Sal* I and *BamH*I restriction sites (underlined).
BAX 1	5′CTAGAAGCACCAAAAAACTGAGCGAATGCCTGAAACGCATTGGC GATGAACTGGATAGCAACATGGCTAGCCTCGAGG3′	Forward. Encodes the Bax BH3 peptide and *Xba* I, *Nhe* I, *Xho* I, and *BamH* I restriction sites (underlined).
BAX 2	5′GATCCCTCGAGGCTAGCCATGTTGCTATCCAGTTCATCGCC AATGCGTTTCAGGCATTCGCTCAGTTTTTTGGTGCTT3′	Reverse. Encodes the Bax BH3 peptide and *Xba* I, *Nhe* I, *Xho* I and *BamH* I restriction sites (underlined).
SCOT 1	5′CTAGAAAACGCCCGGGCGGTGGCGGTGGCAAACGCGG CGGTGGCGGTGGCCCGAAACGCGCTAGCGTCGACG3′	Forward. Encodes the protease OmpT cleavage site sequence and *Xba*I, *Nhe* I, *Sal*I and *BamH*I restriction sites (underlined).
SCOT 2	5′GATCCGTCGACGCTAGCGCGTTTCGGGCCACCGCCAC CGCCGCGTTTGCCACCGCCACCGCCCGGGCGTTTT3′	Reverse. Encodes the protease OmpT cleavage site sequence and *Xba*I, *Nhe* I, *Sal*I, and *BamH*I restriction sites (underlined).

### Oligonucleotide Coupling (Adapters)

**Figure 1:**
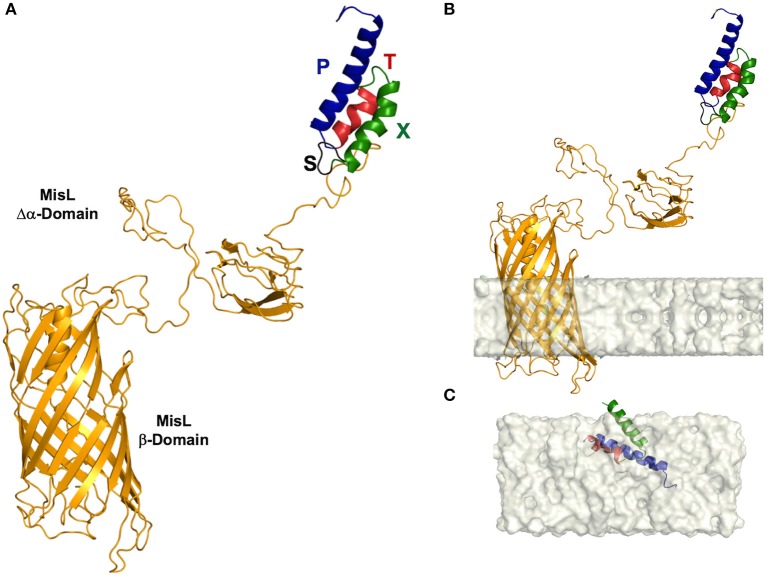
Three-dimensional (3-D) structure of the L-SXTP recombinant protein. **(A)** The MisL autotransporter (L) is observed as a protein composed of two domains. A β-barrel of 12 antiparallel chains (in yellow) and an extracellular α domain that presents to the N-terminal region a combination of several units of secondary structure of β-strands, organized in a specific geometry of β-solenoid architecture. The domains are connected by an α-helical linker, which is embedded within the pore of ~24 Å in diameter of the β-barrel in an extended conformation. The peptides of the fusion core consolidate an α-helical folding and are exposed in the N-terminal region: fusogenic peptide (P) (in blue)-Molecular Tag (T) (in red)-Bax-BH3 peptide (X) (in green)-cleavage site of OmpT protease (S) (in black). **(B)** 3-D structure of L-STXP embedded in a membrane model. We can observe an angle of inclination of 12° with respect to the normal of the membrane and a hydrophobic membrane thickness of 23.8 ± 1.5 Å. Twelve transmembrane segments, in a configuration of antiparallel β-strands, are identified: 1 (312–316), 2 (336–343), 3 (354–362), 4 (385–392), 5 (403–412), 6 (432–443), 7 (452–463), 8 (487–499), 9 (510–520), 10 (545–553), 11 (560–565), 12 (577–584). **(C)** A recreation of the internalization of the fusion core, in a DPPC membrane model, from a theoretical calculation of the membrane internalization propensity of the amino acid residues that constitute the fusion core (data not shown).

The fragments encoding the peptides of interest (adapters) were obtained by coupling 1 pMol of each initiator (forward and reverse) with 200 μM of MgCl_2_ in a volume of 100 μL. The reaction mixture was heated to 94°C for 15 min and slowly cooled to room temperature. The product of coupling contains the encoding sequence of the peptide of interest flanked by the restriction sites Xba I and BamH I, open to ligation without the need for prior digestion, and the internal sites Nhe I, Sal I, Nsi I, Xho I, in accordance with the initiator's design (Figure 1). The primers FP1 and FP2 give origin to the adapter that encodes the fusogenic peptide (P), the primers BAX1 and BAX2 give origin to the adapter that encodes the Bax-BH3 peptide (X), the primers FLAG1 and FLAG2 give origin to the adapter that encodes the Molecular Tag (T), and finally, the primers SCOT1 and SCOT2 give origin to the adapter that encodes the OmpT protease cleavage site (S).

### Plasmid Construction

Following the strategy previously reported by Luria-Perez et al. (34) and Ruiz-Pérez et al. (35), the complete β domain (294 amino acids) and a mutated portion of the α domain (210 amino acids) of the MisL autotransporter were used to express the peptides of interest on the surface of *Salmonella enterica* serovar Typhimurium strain SL3261. From the amino region toward the carboxy terminal, the following peptides were cloned: fusogenic, to destabilize the membrane; Bax BH3, to antagonize the activity of anti-apoptotic proteins from the Bcl-2 family; the molecular Tag Flag, to follow the fusion proteins; and finally, the recognition site of the OmpT protease, to release the peptides from the bacterial surface; this peptide complex was coupled to the mutated amino-terminal of the α domain of MisL (Δα-MisL), followed by the β domain of the MisL (β-MisL) autotransporter. The generated plasmids and the expected fusion proteins are shown in Figure 1A, and the general strategy in plasmid construction is shown in Figure 1B. Plasmid construction was performed in the bacterium *E. coli* DH5-α. Briefly, the plasmid pnirBLTBbMisL (35) was digested with the enzymes Nhe I and BamH I, and a 1,318 bp fragment was obtained, containing the Δα-β domain of the MisL autotransporter; this fragment was purified and stored. The remaining fragment, pnirBLTB (2,627 bp) was used to sequentially insert the adapter fragments encoding the fusogenic peptide (P), the Bax-BH3(X) peptide, the Molecular Tag Flag (T), and the cleavage site of the OmpT protease (S). These adapters contain the external restriction sites Xba I at position 5′ and BamH I at position 3′, open and ready to engage in ligation without the need for prior digestion, and internal sites at position 3′ such as Nhe I, Sal I, Nsi I, or Xho I. Thus, every plasmid adapter may be identified by the loss of an Nhe I site and the gain of new restriction sites at position 3′. Finally, the fragment encoding the Δα-β domain of the MisL autotransporter (L) is inserted. With this strategy, the plasmid that encodes the cell-permeable Bax BH3 peptide that can be expressed and released through the MisL autotransporter system was constructed: pL-SXTP (encoding the Δα-β MisL, the cleavage site of the OmpT protein, the Bax-BH3 peptide, the molecular Tag Flag, and the fusogenic peptide), as well as the control plasmids: pL-XT (encoding the Δα-β MisL, the Bax BH3 peptide, and the molecular Tag Flag), pL-SXT (encoding the Δα-β MisL, the cleavage site of the OmpT protein, the Bax-BH3 peptide, and the molecular Tag Flag), pL-TP (encoding the Δα-β MisL, the molecular Tag Flag, and the fusogenic peptide), pL-XTP (encoding the Δα-β MisL, the Bax-BH3 peptide, the molecular Tag Flag, and the fusogenic peptide). These plasmids gave origin to the recombinant proteins with the same names: L-SXTP, L-XT, L-SXT, L-TP, and L-XTP, respectively. The expression of these recombinant proteins is controlled by the promoter nirB, inducible in anaerobiosis (35).

### Bacterial Culture and Induction Conditions

Bacterial cultures and induction conditions of the recombinant protein were conducted with slight modifications to the previously described methods (34). Briefly, the bacteria *E. coli* DH5-α and *Salmonella enterica* SL3261 were transformed by heat shock or electroporation, respectively, with the previously described plasmids and cultured in broth or Brain Heart Infusion (BHI) agar (Bioxon) with 100 μg/ml ampicillin at 37°C. The *Salmonella enterica* SL3261 strains were cultured in medium supplemented with 0.01% 2,3 dihydroxybenzoic acid (DHB) (Sigma-Aldrich). Considering that recombinant protein expression requires anaerobic conditions, since it is controlled by the induction promoter in anaerobiosis *nirB*, a bacterial colony was cultured in 5 ml BHI broth supplemented with DHB and ampicillin at 37°C and shaken at 200 rpm. When an optic density of 1.0–600 nm was obtained, 100 μl was transferred into a 15-ml tube with thioglycollate broth (Sigma-Aldrich) to promote anaerobic environment, incubated for 10–12 h in DHB with ampicillin, at 37°C and shaken at 200 rpm. The recombinant *Salmonellas* received the name of the recombinant protein they express: *Salmonella enterica* L-SXTP, *Salmonella enterica* L-SXT, *Salmonella enterica* L-XT, *Salmonella enterica* L-SXT, *Salmonella enterica* L-TP, and *Salmonella enterica* L-XTP.

### Recombinant Protein Expression

Recombinant protein expression was analyzed by Western blot and the translocation of the recombinant proteins to the bacterial surface by immunofluorescence and flow cytometry.

#### Western Blot Assays

Recombinant protein expression was analyzed with slight modifications to the previously described techniques (34, 35). Briefly, recombinant bacteria, after induction as previously described, were harvested by centrifugation, and 10^9^ bacteria were resuspended in 100 μl loading buffer (Tris 0.5 M, pH 6.8, SDS 2%, 2-mercaptoethanol 140 mM, and bromophenol blue 0.1%) and boiled at 94°C for 15 min. Recombinant proteins were analyzed by electrophoresis on 12.0% acrylamide gels under reduction conditions (SDS-PAGE), and the Western blots (WB) were performed as previously described (34). Briefly, the transfer of proteins to the nitrocellulose membrane was conducted by semi-dry transference at 25 V and for 10 min in the Trans-Turbo Blot System (Bio-Rad). Immunodetection of the recombinant proteins was performed with a monoclonal antibody induced in an anti-Flag mouse (1 mg/ml, Sigma-Aldrich), diluted 1:1,000 in PBA 1× (1% BSA in PBS 1×). The membranes were incubated for 2 h with the primary antibody and subsequently for 1 h with the anti-mouse IgG-HRP secondary antibody (1 mg/ml, Abcam) at room temperature, diluted 1:1,000 in PBA 1×. Finally, the membranes were developed in a solution of 4-chloro-α-naphtol (Sigma-Aldrich) in methanol-PBS 1×, pH 7.4, and hydrogen peroxide (J.T. Baker).

#### Immunofluorescence and Flow Cytometry Assays

Recombinant protein translocation to the bacterial surface via the MisL autotransporter was evaluated by immunofluorescence and flow cytometry, as previously described (34, 35). Briefly, after inducing the expression of the recombinant proteins as described above, 10^8^ bacteria were incubated with antibody mouse anti-Flag FITC (1 mg/ml, Sigma-Aldrich) at a dilution of 1:100 in PBA 1× for 2 h, at room temperature, at 100 rpm, in dark conditions. The bacterial suspension was washed in PBS 1× and resuspended in 50 μl PBS 1×, 5 μl of which were used for fluorescent microscopy analysis (Olympus Microscope, model IX73), and the rest was resuspended in 450 μl PBS 1× for flow cytometry analysis in the CytoFLEX system (Beckman Coulter).

### Cell Lines and Cell Cultures

The Ramos cell line (Burkitt's lymphoma), a human B NHL cell line, was obtained from the American Type Cell Collection (ATCC, CRL-1923) and cultured in Advanced RPMI 1640 medium (Invitrogen), supplemented with 1% antibiotics-antimycotics containing 10,000 U/ml penicillin G, 10 mg/ml streptomycin, and 25 μg/ml amphotericin B and 4% fetal bovine serum (FBS, Invitrogen). Cultures were permanently maintained at 37°C and 5% CO_2_. For the infection assays, the cells were grown with Advanced RPMI 1640 medium (Invitrogen), supplemented with 2% FBS without antibiotics (Invitrogen).

### Detection of the Anti-apoptotic Molecules by Western Blot

The detection of the anti-apoptotic molecules was conducted with slight modifications to the technique described by Hernández-Luna et al. (16). A million Ramos cells were lysed with RIPA lysis buffer (Sigma-Aldrich) supplemented with a cocktail of protease inhibitors (Roche). Protein quantification was performed with the bicinchoninic acid kit by ThermoFisher Scientific. For the Western blot, 25 μg of total protein was placed in each well, and an electrophoresis on 12% polyacrylamide-SDS gel was performed. The proteins were then transferred to nitrocellulose membranes (Bio-Rad) with a Trans-Turbo Blot System by Bio-Rad (25 V, 10 min). For the detection of the Bcl-_XL_ and Mcl-1 proteins, we used anti-Bcl-_XL_ and anti-Mcl-1 antibodies (Cell Signaling) induced in rabbits and diluted 1:1,000 in blocking buffer (Li-Cor), and as a secondary antibody, goat anti-rabbit IgG IR Dye 680 cw (Li-Cor) diluted 1:10,000 in blocking buffer (Li-Cor). As a constitutive protein control, we used an anti-β tubulin antibody induced in rabbit and diluted at 1:1,000 in blocking buffer (Li-Cor). Finally, the image was obtained and analyzed in the system for Infrared Fluorescent Imaging, Odyssey CLx (Li-Cor).

### Tumor Cell Infection With Recombinant *Salmonella* Strains

Infection of tumor cells with recombinant *Salmonella* strains was conducted as described by Vendrell et al. (53). Briefly, Ramos cells were cultured in 48-well plates (500,000 cells/well) in 1 mL of Advanced RPMI 1640 medium supplemented with 2% FBS without antibiotic. The cells were infected at a Multiplicity of Infection (MOI) of 100 with previously induced recombinant bacteria. After centrifugation at 2,000 rpm for 5 min to foster the interaction between bacteria and cells, they were incubated at 37°C and 5% CO_2_ for 2–10 h. Subsequently they were washed twice in base advanced RPMI 1640 medium supplemented with gentamycin (Sigma-Aldrich) 100 μg/mL and finally resuspended in 1 mL of advanced RPMI 1640 medium supplemented with 2% FBS and 50 μg/mL gentamycin and used in the cellular viability and apoptosis detection protocols. Vincristine (Sigma-Aldrich) 0.5 nM diluted in injectable water was used as a positive control in all cases; this drug is used as treatment for NHL.

### Cellular Viability Assay by Trypan Blue Dye Exclusion

The viability assays were conducted with slight modifications to the previously described technique (53). Briefly, 500,000 Ramos cells, infected at a MOI of 100 with the previously induced recombinant *Salmonella* strains expressing the peptides of interest via the MisL autotransporter, were incubated for 2–10 h at 37°C and 5% CO_2_ and analyzed in an automated cell counter (BioRad) to determine cell viability by trypan blue dye exclusion (Invitrogen). Vincristine (Sigma-Aldrich) 0.5 nM diluted in injectable water was used as a positive control.

### Apoptosis Assays

The *in vitro* apoptosis assays were analyzed by flow cytometry and Western blot as follows:

a). Determination of Active Caspase-3 Cells and TUNEL by Flow Cytometry. These assays were performed with modifications to the technique described by Hernández-Luna et al. (16, 33). After treating the Ramos cells (500,000 cells) with the induced recombinant *Salmonella* strains (MOI of 100) during 8 h, cells were washed, fixed, permeabilized (Cytofix/Cytoperm, Becton Dickinson) and stained with the anti-active caspase-3 FITC antibody according to the FITC Active Caspase-3 Apoptosis Kit (BD Pharmingen) instructions; for the TUNEL assay, the enzyme and substrate were added according to the instructions of the *in situ* Cell Death Detection Kit, Fluorescein (Roche, Sigma-Aldrich). Data acquisition and analysis were performed in a CytoFLEX (Beckman Coulter) flow cytometer.

b). Determination of Caspase-3 and Cleaved PARP by Western Blot. The detection of the apoptotic molecules was conducted with slight modifications to the technique described by Hernández-Luna et al. (16). After treating the Ramos cells (6 × 10^6^ cells) with the induced recombinant *Salmonella* strains (MOI of 100) during 8 h, cells were lysed with RIPA lysis buffer (Sigma-Aldrich) supplemented with a cocktail of protease inhibitors (Roche). Protein quantification was performed with the bicinchoninic acid kit by ThermoFisher Scientific. For the Western blot, 15 μg of total protein was placed in each well, and an electrophoresis on 15% polyacrylamide-SDS gel was performed. The proteins were then transferred to nitrocellulose membranes (Bio-Rad) with a Trans-Turbo Blot System by Bio-Rad (25 V, 10 min). For the detection of the complete caspase-3 and cleaved PARP proteins, we used an anti-caspase 3 (Human specific, Abcam) and anti-cleaved PARP-1 (Human specific, Cell Signaling) antibodies induced in rabbits and diluted 1:300 and 1:500, respectively, in blocking buffer (Li-Cor), and as a secondary antibody, goat anti-rabbit IgG IR Dye 680 cw (Li-Cor) diluted 1:10,000 in blocking buffer (Li-Cor). As a constitutive protein control, we used an anti-β tubulin antibody (Abcam), induced in rabbit and diluted at 1:7,000 in blocking buffer (Li-Cor). Finally, the image was obtained and analyzed in the system for infrared fluorescent imaging, Odyssey CLx (Li-Cor).

### Mice

Pathogen-free, female athymic BALB/c *nu/nu* mice between the ages of 6 and 8 weeks were obtained from the bioterium of the National Institute of Medical Sciences and Nutrition Salvador Zubiran and kept in an enriched and sterile environment in the bioterium of the Children's Hospital of Mexico Federico Gomez in accordance with the local and federal regulations for the Care and Use of Laboratory Animals. Distress and suffering were avoided as much as possible. All procedures were supervised by a clinical veterinarian.

### Murine Xenograft Model

The development of the mouse xenograft model of the human B NHL was performed with slight modifications to the previously described method by Manders et al. (54). Briefly, female athymic BALB/c *nu/nu* mice between 6 and 8 weeks of age were inoculated intraperitoneally with cyclophosphamide (100 mg/kg of body weight), and 24 h later, they were anesthetized in a gas chamber with isoflurane, 100 units (Abbott); they were then subcutaneously inoculated on the right flank with 10^7^ Ramos cells in a volume of 100 μl of Opti-MEM reduced serum medium without phenol red (Invitrogen). As soon as the tumors were visible and reached an approximate size of 100–150 mm^3^ (approximately after 15 days of inoculation), mice were separated into groups of five to evaluate the antitumor activity of the recombinant *Salmonellas*. The volume of the ellipsoidal tumor was determined with the following formula: volume = 1/6π X length X width X height.

### Antitumor Activity of the Recombinant *Salmonella* Strains

These assays were performed with slight modifications to the technique described by Nakase et al. (55). Briefly, groups of five mice with tumors measuring ~100–150 mm^3^ received four doses in the tail vein, at intervals of 7 days, of 100 μl with 1 × 10^7^ CFU in PBS 1× from each of the different previously induced recombinant *Salmonella* strains. The mice were also administered with 0.6 mg/ml ampicillin in their drinking water. Control mice received 100 μl PBS 1× following the same inoculation protocol. Tumor size was measured with a Vernier caliper and calculated as mentioned previously. Survival was registered daily. After 5 days of last inoculation of recombinant *Salmonella* strains, the mice were euthanized, and the tumor tissue was resected and stored for bacterial culture and immunohistochemistry assays. Serums were collected for inflammatory cytokine determination.

### Bacterial Culture From Tumor Tissue: Determination of Colony-Forming Units (CFU)

To demonstrate recombinant *Salmonella* strains tumor targeting, the murine xenograft model of the human B NHL was treated with the recombinant *Salmonella* strains, and then the bacteria were isolated from the tumor tissue; this procedure was performed with slight modifications to the previously described method by Miyake K et al. (56). Briefly, groups of three mice with tumors measuring ~100–150 mm^3^ received four doses in the tail vein, at intervals of 7 days, of 100 μl with 1 × 10^7^ CFU in PBS 1× from each of the different previously induced recombinant *Salmonella* strains. Mice were also administered with 0.6 mg/ml ampicillin in their drinking water. Control mice received 100 μl PBS 1× following the same inoculation protocol. Five days after last inoculation, mice were euthanized, and the tumors were resected for bacteria culture. The tumor specimens were weighed, homogenized, and suspended in 1 ml PBST (0.05% Tween 20 in PBS 1×). The suspension was diluted five times each up to 1:10,000, then cultured in BHI agar medium in the presence or absence of ampicillin (100 μg/ml) for 12 h. Finally, the colonies were counted and reported as CFU per tissue gram (CFU/g). We also analyzed the presence of the bacteria in the tumor tissue, performing an immunohistochemical assay using as primary antibody an anti-*Salmonella* induced in rabbit.

### Detection of Apoptotic Markers in Tumor Tissue by Immunohistochemistry

To demonstrate that the antitumor activity of the recombinant *Salmonella* strains was mediated by an apoptosis mechanism, tumor-bearing mice that previously received four doses in the tail vein of the recombinant *Salmonella* strains, as described previously, were euthanized after 5 days of the last inoculation, and the tumors were resected for immunohistochemical staining of apoptotic markers.

#### Tumor Histology

The tumors were removed and fixed with absolute ethanol. The tumors were dehydrated and then embedded in paraffin. For histological examination, 4-μm-thick sections were cut on a semi-automated microtome (Leica, USA), placed on glass slides, and deparaffinized. The slides were stained sequentially with hematoxylin and eosin (H&E) to assess the tumor histology. Slides were analyzed under an Olympus BX-40 microscope.

#### Determination of Apoptotic Markers by Immunohistochemistry Staining

The expression of active Caspase-3, Caspase-8, Ki67, and the presence or absence of *Salmonella* were analyzed in 4-μm tumor slices by immunohistochemistry using specific antibodies as previously described (57). In brief, antigen retrieval was performed by immersing the slides in Antigen Unmasking Solutions Citrate-Based (VECTOR) for 20 min into pressure cooker. Endogenous peroxidase activity was inhibited by immersing the slides in 3% H_2_O_2_-methanol for 15 min two times, and background nonspecific binding was reduced by incubating with normal horse serum in PBS 1× for 60 min. The slides were incubated 60 min at room temperature with antibody against active Caspase-3 (GeneTex, dilution 1:250), Caspase 8 (GeneTex, dilution 1:1,500), Ki67 (BioSystems, dilution 1:100), and *Salmonella* (1:4000). Finally, the slides were washed two times in PBS 1× 0.1 M pH 7.4 for 5 min. In order to reduce variability. All samples were processed at the same time in a single experiment using a single batch of antibody. After washing, the slides were incubated with the ImmPRESS HRP anti-rabbit IgG polymer system for 30 min at room temperature and then with 3,3′-diaminobenzidine tetra-hydrochloride (DAKO, Carpinteria, CA, USA) for 1–5 min. The reaction was arrested with distilled water, and the slides were counterstained with hematoxylin. Thereafter, the tissues were washed in tap water for 5 min, dehydrated sequentially in 70, 90, and 100% ethanol and xylene and then mounted.

#### Detection of Apoptosis by Terminal Deoxynucleotidyl Transferase dUTP Nick End Labeling (TUNEL) Assay in Tumor Tissue

The DNA fragmentation in tumor tissue was evaluated by *in situ* TUNEL assay using an *in situ* Cell Death Detection Kit (POD) (Roche Applied Science, Mannheim, Germany) following the manufacturer's instructions in 4-μm tumor slices. Briefly, an Antigen Unmasking Solution Citrate-Based (VECTOR), incubated in a water bath for 20 min, was used for antigen retrieval, and endogenous peroxide activity was blocked with methanol and 3% hydrogen peroxide for 15 min. They were blocked with normal horse serum for 1 h, and the preparations were incubated for 60 min with TUNEL reaction mixture [Terminal transferase (Tdt) diluted in a buffer solution that included fluorescein conjugated oligonucleotides] and subsequently incubated with an anti-fluorescein antibody (Converter-POD) for 30min, both reactions were performed at 37°C in a humidified atmosphere in the dark. Color was generated by adding the substrate 3,3′-diaminobenzidine (DAB) (VECTOR) for 1–2 min, and counterstaining was performed with hematoxylin. The tissues were dehydrated and covered with resin. Finally, the slides were analyzed under light microscopy (Olympus BX-40), and the nuclear positive cells were determined.

In all cases, for digital automated morphometry, the immunohistochemically stained sections were digitizing at 40× magnification using an Aperio Scanscope CS (Aperio, Vista, CA). The images were reviewed using an ImageScope (Aperio). Once the areas were recorded, they were sent for automated image analysis using the Spectrum Software V11.1.2.752 (Aperio). For the within-tissue intensity, an algorithm was developed to quantify the total protein expression. The output from the algorithm gives a number of quantitative measurements, namely, the intensity of positive staining. The staining intensity was categorized as 0 (no staining), 1+ (weak), 2+ (moderate), and 3+ (strong).

#### Determination of Inflammatory Cytokines by Cytometric Bead Array (CBA)

After the antitumor activity assays of the murine model of NHL xenotransplantation induced by different recombinant strains of *Salmonella enterica*, as it was previously described, the mice were euthanized, and the serum samples obtained were analyzed by means of the CBA in order to characterize the profile of inflammatory cytokines induced along the treatment. The serum samples were prepared according to the BD™ CBA Mouse Inflammation Kit (Becton Dickinson, BD) specifications. In this assay, six bead populations with distinct fluorescence intensities have been coated with capture antibodies specific for Interleukin-6 (IL-6), Interleukin-10 (IL-10), Interleukin-12p70 (IL-12p70), Monocyte chemoattractant protein-1 (MCP-1); Interferon-γ (IFN-γ), and Tumor necrosis factor-α (TNF-α) proteins. The six bead populations are mixed together to form the BD CBA, which is resolved in a red channel (i.e., FL3 or FL4) of a flow cytometer. The captured beads, PE-conjugated detection antibodies, and recombinant standards or test samples are incubated together to form sandwich complexes. Following the acquisition of sample data using the flow cytometer, the sample results are generated in graphical and tabular format using the BD CBA Analysis Software or FCAP Array™ Software. Briefly, the standards dilutions and the undiluted serums were exposed to a mixture of cytokines (IL-12p70, IL-6, IL-10, TNF-α, IFN-γ, MCP1) together with the PE-coupled detection reagent. The mixture was incubated for 2 h at room temperature in darkness. After that, it was washed with 1 ml of washing buffer and centrifuged at 200 g for 5 min to then remove the supernatant and recovering with 100 μl of washing buffer. The samples were finally analyzed with a BD FACS Canto II flow cytometer, resulting in a total of 500 events per sample. The FCAP Array software of BD (3.0 version) was used for the quantitative analysis of the concentration and Median Fluorescence Intensities (MFI) of each cytokine.

### Statistical Analysis

To determine the differences between cells or mice groups treated with the different recombinant *Salmonella* strains, we used one-way analysis of variance (ANOVA) and *post hoc* Bonferroni tests, with a 95% confidence interval. In all cases, the average of three or more independent experiments is presented ± the standard deviation (*SD*). Animal survival was analyzed with the log-rank test on Kaplan-Meier curves. Differences were considered significant at *p* ≤ 0.05 in all comparisons. Statistical analysis was performed with GraphPad Prism 5 software.

## Results

### 3-D Structure of the MisL Autotransporter System Carrying the Cell-Permeable Bax BH3 Peptide

Type V autotransporter proteins, the family MisL belongs to, present a diverse variety of functions such as cell adhesion, pathogenesis phenomena, or mediating in the immune response interruption of the host. This is the result of having a rich structural diversity given by its large amount of secondary structures, the geometrical organization of their domains, and the different oligomerization states that these proteins arrange. In our case, because of its function, MisL-SXTP chimera (L-SXTP) has been clearly identified as a molecular carrier. Therefore, and since the fused peptides are located toward the N-terminal region, characterizing the 3-D structure of L-SXTP together with the evaluation of the effect that these peptides may have on its folding and stability are extremely relevant. With this motivation, molecular modeling of the L-SXTP chimera allowed us to obtain a good approximation of the 3-D structure of each of the components of the chimera introduced in this work (Figure 1A). The MisL autotransporter is observed as a protein composed of two domains. A first β-barrel domain made up of 12 antiparallel chains with five short handles is internalized toward the periplasmic space, and a second extracellular domain showing at the N-terminal region a combination of several units of β-strands was organized in a specific geometry known as β-solenoid architecture. Inside this former structural motif, chains of amino acids are densely packed in a compact core that was predominantly hydrophobic. Finally, connecting both domains, an α-helical linker extends along the first domain β-barrel pore of ~24 Å diameter. Although the peptides of the fusion core consolidate an α-helical folding and the cleavage site of the OmpT protease is revealed as a flexible loop, these motifs do not induce any unfolding or instability of the 3-D structure of MisL, neither in its global configuration, nor in the proximity to the β-solenoid (Figure 1A). The fusion core is exposed in the N-terminal region, and it is the cleavage site of the OmpT protease that facilitates its release by the PgtE protease (data not shown) (58). When predicting the spatial arrangement of our model in a lipid bilayer, a 12° tilt angle with respect to the normal of the membrane can be observed. In addition, 12 transmembrane segments can be identified in a configuration of antiparallel β-strands [1 (312–316), 2 (336–343), 3 (354–362), 4 (385–392), 5 (403–412), 6 (432–443), 7 (452–463), 8 (487–499), 9 (510–520), 10 (545–553), 11 (560–565), 12 (577–584)] embedded in a hydrophobic membrane thickness of 23.8 ± 1.5 Å (Figure 1B). The depth of penetration of these structures in the membrane coincides with those reported for other 3-D structures of various autotransporters deposited in the protein data bank. With respect to the fusion core [Tag peptide (T) bound to Bax BH3 peptide (X) and the fusogenic peptide (P)], we hypothesize that after being excised due to proteolytic processing cleavage site of the OmpT, it is released with a helix-loop-helix conformation. When making a theoretical prediction about the internalization of the core in the membrane, we observed that given its amino acid composition and its hydrophobicity, it is possible that it crosses the membrane through a diffusion process (Figure 1C).

### Construction of the Plasmids *pL-XT, pL-SXT, pL-TP, pL-XTP*, and *pL-SXTP*

**Figure 2:**
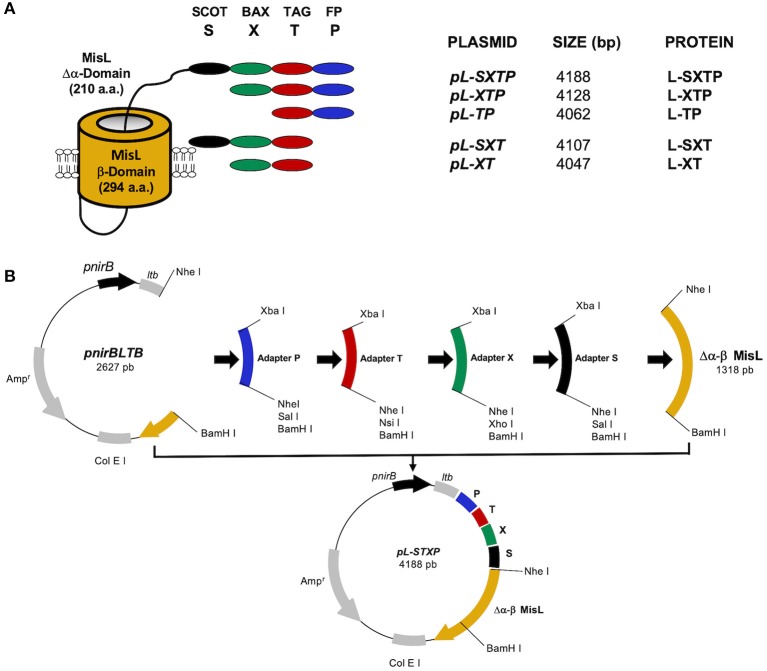
Construction of the recombinant proteins. **(A)** Representative scheme of the expressed and released peptides from the surface of *S. enterica* SL3261 through the misL autotransporter. The β domain of MisL is constituted by 294 amino acids, and the mutated α domain contains 210 amino acids, the peptides of interest are linked to the N terminal portion of the mutated α domain. **(B)** General strategy for the construction of the plasmids. The sequences that encode for the fusogenic peptide (P), the molecular Tag flag (T), the Bax BH3 peptide (X), and the cleavage site for the OmpT protease (S) were inserted in the pnirLTBβMisL plasmid, which contains the signal peptide of heat-labile enterotoxin B subunit (LTB) of enterotoxigenic *E. coli* under the anaerobiosis inducible *nirB* promoter. The sequence of the β MisL autotransporter was extracted from the pnirLTBβMisL plasmid by a double digestion with the Nhe I and BamH I enzymes; this fragment was purified and stored. Later, the adapters that encode for each peptide were inserted in a sequential manner in the *pnirBLTB* +*P*+*T*_…_*n* plasmid, and finally, the fragment that encodes for the β domain of the MisL autotransporter was reinserted.

The ability of the MisL autotransporter to translocate the proteins on the surface of *Salmonella enterica* SL3261 (*S. enterica* SL3261) and its release from it to the tumoral microenvironment has been previously reported by our group (34, 35, 58). In this work, we designed recombinant proteins, controlled by the promoter *nirB*, inducible in a microaerophilic environment; these proteins contain in their amino terminal portion the signal peptide of heat-labile enterotoxin B subunit (LTB) of enterotoxigenic *E. coli*, necessary to perform the translocation to the periplasmic space through the Sec Dependent System (59). According to the described strategy for the construction of the plasmids, the adapters that encode for each one of the peptides were inserted sequentially downstream from the *nirB* promoter's sequence and the signal peptide LTB sequence, as shown in Figure 2B. For the plasmid that encodes the cell-permeable Bax BH3 peptide, which expresses and releases from the MisL autotransporter system, it was first inserted in the adapter that encodes the fusogenic peptide(P) to destabilize the membrane, followed by the adapter for the Bax BH3 peptide (X) to antagonize the activity from the anti-apoptotic proteins of the Bcl-2 family, subsequently, the molecular Tag Flag (T) for the tracking of the recombinant proteins, and finally, the cleavage site of the protease OmpT (S) to release the peptides from the bacterium surface; this peptide complex was linked to the sequence that encodes for the autotransporter Δα-β MisL (L), and the plasmid received the name *pL-STXP* (4,188 bp) (see Figure 2B). Under this same strategy, four plasmid controls were built: *pL-XT* (4,047 bp), which encodes for the Bax BH3 peptide; *pL-SXT*(4,107 bp), which encodes for the cleavage site of the OmpT protease and the Bax BH3 peptide; *pL-TP*(4,062 pb), which encodes for the fusogenic peptide; *pL-XTP* (4,128 pb), which encodes for the Bax BH3 peptide and the fusogenic peptide; all cases encode for the molecular Tag Flag and the autotransporter Δα-β MisL. All of the constructions were sequenced. These plasmids originated from the recombinant proteins with the same names: L-SXTP, L-XT, L-SXT, L-TP, and L-XTP, respectively (Figure 2A).

### The Cell-Permeable Bax BH3 Peptide Is Expressed on the Surface of the *S. enterica* Through the MisL Autotransporter System

**Figure 3:**
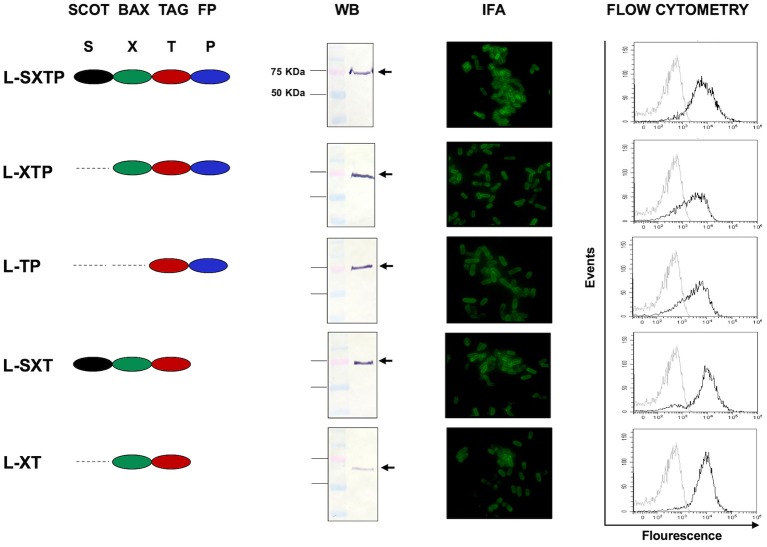
Expression of the recombinant proteins on the surface of *S. enterica*. The expression and translocation of the recombinant proteins on the surface of *S. enterica* was evaluated after 12 h of induction in a thioglycollate medium. From left to right, the Western blots (WB), performed with total bacteria extracts, showed a protein of ~75 KDa. The immunofluorescence (IFA) and the flow cytometry were performed with bacterial suspensions. Controls in the flow cytometry (histogram to the left) are *S. enterica* SL3261 expressing only the autotransporter MisL. In all assays, the immunodetection was performed with monoclonal anti-Flag antibody.

The expressions of the recombinant protein L-SXTP (express and release the Cell-permeable Bax BH3 Peptide through MisL autotransporter) and the control proteins L-XT, L-SXT, L-TP, and L-XTP were evaluated by Western blot using total extracts obtained from the bacteria *E. coli* DH5-α and *S. enterica* SL3261 that were previously transformed with the different plasmids and cultured in anaerobic conditions during 10–12 h. The Western blot using an anti-Flag antibody revealed the expected protein of ~75 KDa. The inmunofluorescence (IFA) and the flow cytometry showed that all recombinant proteins were expressed on the surface of *S. enterica* SL3261 with a similar expression profile. Transformed *Salmonella* with the different plasmids received the name of the recombinant protein that they expressed (Figure 3). In order to reinforce the evidence on the cell-permeable Bax BH3 peptide expression over the *S. enterica* surface through the MisL autotransporter, the *S. enterica* SL3261 (negative control), the *S. enterica* SXTP (containing the cell-permeable Bax BH3 peptide coding plasmid without the MisL autotransporter; thus, only expressed in the cytosol), and the *S. enterica* L-SXTP (containing the cell-permeable Bax BH3 peptide coding plasmid expressed in the surfaced by means of the MisL autotransporter), strains were induced as described previously. After that, the protein expression was evaluated by DotBlot, flow cytometry, and immunoelectron microscopy; in all cases, using a mouse anti-Flag antibody (Supplementary Figure 1). As expected, the presence of cell-permeable Bax BH3 peptide, 6 KDa approx., was detected by DOT Blot in the *S. enterica* SXTP strains extracts. And the presence of cell-permeable Bax BH3 peptide coupled to the MisL autotransporter, 75 KDa approx., was detected for *S. enterica* L-SXTP (Supplementary Figure 1A). The cell-permeable Bax BH3 peptide expression in the bacterial surface was analyzed by means of flow cytometry in non-permeable conditions. Resulting in an absence of peptides detected over the surface of *S. enterica* SXT given that it lacks the translocation machinery, *S. enterica* SL3261 was used as negative control. The translocation effect is acquired with the genetic coupling to the MisL autotransporter, as it can be observed in the right shift of the *S. enterica* L-SXTP histogram (Supplementary Figure 1B). To better confirm this observation, immunoelectron microscopy was performed with the recombinant *Salmonellas*. Mouse anti-Flag antibodies were used as the primary antibody, and anti-mouse IgG antibodies coupled to 20 nm gold particles were used as the secondary antibody. The cross sections of bacteria show that the cell-permeable Bax BH3 peptide was only detected on the bacterial surface when coupled to the MisL autotransporter (*S. enterica* L-SXTP). In this former case, the peptide is also detected in the bacterial cytosol as well as in the case of *S. enterica* SXTP and *S. enterica* L-SXTP, as expected (Supplementary Figure 1C).

### The Cell-Permeable Bax BH3 Peptide Expressed and Released From *S. enterica* Induces Cell Death of NHL Cells

**Figure 4:**
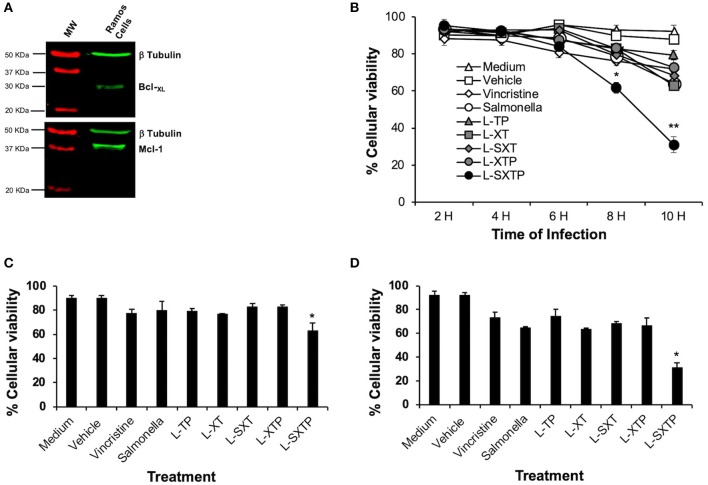
*S. enterica* L-STXP reduces the viability of Ramos cells. **(A)** Expression of the anti-apoptotic proteins Bcl-_XL_ and Mcl-1 in Ramos cells (Burkitt lymphoma, NHL). Proteic extracts of Ramos cells (25 μg per well) were analyzed by Western blot using the anti Bcl-_XL_, anti Mcl-1, and anti β-tubulin antibodies diluted to 1:1,000. As secondary antibody, we used goat antirabbit IgG antibody (IRDye Oddissey) 1:10,000. **(B)** Decrease in the Ramos cells viability to treatment with *S. enterica* L-STXP. Ramos cells were infected to a MOI of 100 with the different recombinant *Salmonella* strains (L-TP, L-XT, L-SXT, L-XTP, and L-SXTP) during 2–10 h. As controls, we used non-treated cells (medium) as a negative control, sterile water as solvent to vincristine (vehicle), non-transformed *S. enterica* SL3261 (*Salmonella*), and vincristine 0.5 nM as a positive control. The viability was analyzed by trypan blue dye exclusion. A representative experiment of the viability of Ramos cells at 8 **(C)** and 10 h **(D)** after the infection with the recombinant *Salmonella* strains confirms the dramatic reduction of the viability of Ramos cells treated with recombinant *Salmonella* L-SXTP compared with controls. In the graphics, the error bars represent the average ± *SD* of measurements in triplicate. The results are representative of three independent experiments. ANOVA test was performed with Bonferroni *post hoc* for the difference between the groups. For **(B)**, *p < 0.001, **p < 0.0001; for **(C)**, *p < 0.001; for **(D)**, *p < 0.0001.

After confirmed expression of the cell-permeable Bax BH3 peptide on the surface of the *S. enterica* L-STXP, and the other controls, we analyzed its effect over the viability of NHL cells. Accordingly, Ramos cells that express anti-apoptotic proteins as Bcl-_XL_ and Mcl-1 (Figure 4A) were infected to a MOI of 100, during 2, 4, 6, 8, and 10 h with the recombinant *Salmonella* strains, previously transformed with the different plasmids and cultured in anaerobic conditions during 10 h. As additional controls, we included non-transformed *S. enterica* SL3261 and vincristine 0.5 nM (drug employed as chemotherapy in NHL). After the infection time, the cell viability assays was analyzed by trypan blue exclusion. The Figure 4B shows that *S. enterica* L-SXTP, which expresses and releases the cell-permeable Bax BH3 through the MisL autotransporter, reduced dramatically the cellular viability after 8 h of infection to values of 61% ± 2.8, and this effect was greater 10 h after infection (31% ± 4.3) compared with the non-treated cell control (medium), which remained with viability values above 95% ± 0.5 at all the analyzed times. The treatment with non-transformed *S. enterica* SL3261 reduced the viability of Ramos cells to a 79% ± 4.3 at 8 h and to 64% ± 1.4 at 10 h; these values were very similar to the ones obtained with the *S. enterica* L-XT treatment (82.6% ± 2.3 at 8 h and 63% ± 1.4 at 10 h) and *S. enterica* L-SXT treatment (79.6% ± 1.5 at 8 h and 68.3% ± 1.5 at 10 h). A lesser effect over the viability was observed when the cell was treated with *S. enterica* L-XTP (83.3% ± 0.5 at 8 h and 72.6% ± 7.0 at 10 h) and *S. enterica* L-TP (83% ± 2.6 at 8 h and 79.3% ± 2.5 at 10 h). Vincristine 0.5 nM reduced the viability of Ramos cells to values of 76.3 ± 2.5% at 8 h and to 72 ± 2.0% at 10 h of treatment. The control for the vehicle (water) used to dissolve vincristine showed viability values of 91% ± 2.0 at all analyzed hours in the kinetics. Figure 4C shows a representative experiment of the viability of Ramos cells at 8 h after the infection with the recombinant *Salmonella* strains. These results were consistent with the observed data at 8 h of infection in the kinetics: medium and vehicle maintained the celular viability at 90% ± 2.2 values; vincristine 0.5 nM, non-transformed *S. enterica*, and the *Salmonellas* L-XT, L-SXT, L-TP, and L-XTP affected the viability to values from 76.3% ± 1.0 to 82.6% ± 2.7, and the significant reduction in the viability was confirmed when the cells were treated with *S. enterica* L-SXTP (62.6% ± 6.7). Figure 4D shows a representative experiment of the viability of Ramos cells at 10 h after the infection with the recombinant *Salmonella* strains. These results were also consistent with the observed data at 10 h of infection in the kinetics: medium and vehicle mantained the cellular viability at 93.5% ± 0.6 values; vincristine 0.5 nM, non-transformed *S. enterica*, and the *Salmonellas* L-XT, L-SXT, L-TP, and L-XTP affected the viability to values from 74.0% ± 5.8 to 67.0% ± 8.1, and the dramatic reduction in the viability was confirmed when the cells were treated with *S. enterica* L-SXTP (33.5% ± 0.5). These data demonstrated that the cell-permeable Bax BH3 peptide, expressed and released by *S. enterica* through the MisL autotransporter, may kill NHL cells.

### The Cell-Permeable Bax BH3 Peptide Expressed and Released From *S. enterica* Promotes Apoptosis of NHL Cells

**Figure 5:**
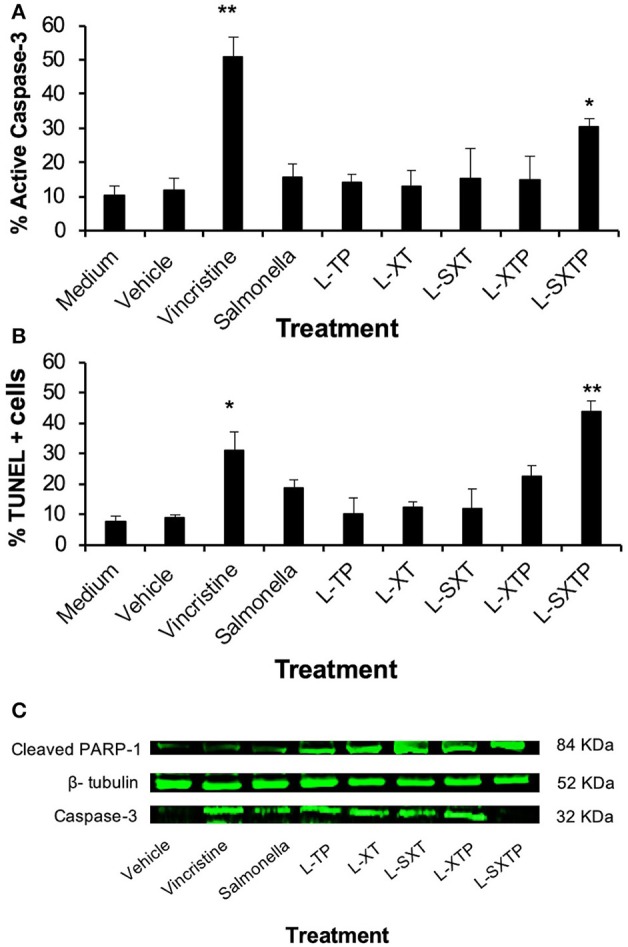
*S. enterica* L-STXP induces the apoptosis of Ramos cells. Ramos cells were infected during 8 h to a MOI of 100 with the different recombinant *Salmonella* strains (L-TP, L-XT, L-SXT, L-XTP, and L-SXTP). As controls, we used non-treated cells (medium) as a negative control, injectable water as solvent to vincristine (vehicle), non-transformed *S. enterica* SL3261 (*Salmonella*) and vincristine 0.5 nM as a positive control. The apoptosis was analyzed by determination of caspase-3-active **(A)** and TUNEL-positive cells **(B)**. **(C)** Infected tumor cells were lysed, and total protein was obtained. The expression of caspase-3 and PARP-1 was examined by Western blotting. β-tubulin was used as the loading control. In the graphics, the error bars represent the average ± *SD* of three independent experiments. ANOVA test was performed with Bonferroni *post hoc* for the difference between the groups. In all cases, *p < 0.001, **p < 0.0001.

In order to determine whether the reduction of the Ramos cells viability, mediated by *S. enterica* L-SXTP, was due to cellular death by apoptosis, promoted by the cell-permeable Bax BH3 peptide, and expressed and released of the MisL autotransporter, Ramos cells were treated during 8 h with the recombinant *Salmonella* strains (MOI of 100) transformed with the different plasmids and cultured in anaerobic conditions for 10 h. As additional controls, we included non-transformed *S. enterica* SL3261 and vincristine 0.5 nM. After the infection, the cells were assessed to determine the caspase-3 activation and DNA fragmentation (TUNEL). As shown in Figure 5A, Ramos cells treated with *S. enterica* L-SXTP showed a greater number of active caspase-3 cells (30.4% ± 2.3) compared with the non-transformed *S. enterica* SL3261 controls (16.7% ± 3.8) and the recombinant *S*. strains L-XT (12.3% ± 4.0), L-SXT (13.4% ± 7.9), L-TP (14.7% ± 1.9), and L-XTP (13.7% ± 6.0). High values were also observed for the positive control of vincristine (49.5% ± 5.8) compared with the medium (10.3% ± 2.9) and vehicle controls (11.7% ± 3.6) as expected. These results were consistent with the data from the TUNEL assay, in which Ramos cells treated with *S. enterica* L-SXTP showed up to 51.7% ± 4.0 of TUNEL-positive cells compared with the non-transformed *S. enterica* SL3261 controls (25.9% ± 3.8) and the recombinant *Salmonellas* L-XT (15.9% ± 1.9), L-SXT (20.9% ± 6.7), L-TP (16.5% ± 5.1), and L-XTP (24.9% ± 3.8). In this case, great values of TUNEL-positive were also observed for the positive control of vincristine (31.3% ± 6.2) compared with the medium (9.5% ± 1.9) and vehicle (9.3% ± 1.1) (Figure 5B). Similar results were obtained with a TUNEL assay using immunocytochemical staining (Supplementary Figure 2). Another characteristic event of apoptosis is the proteolytic cleavage of poly (ADP-ribose) polymerase-1 (PARP-1), a nuclear enzyme involved in DNA repair, DNA stability, and transcriptional regulation. Particularly, caspase-3 and caspase-7 cleave the 116 KDa form of PARP-1 and generate an ~89 and 24 KDa fragment. The cleavage of PARP-1 between Asp214 and Gly215 results in the separation of the two zinc-finger DNA-binding motifs from the automodification and catalytic domains, thus preventing the recruitment of the enzyme to sites of DNA damage. Cleaved PARP-1 has been considered as a hallmark of apoptosis (60). In Figure 5C, we analyzed by Western blot the presence of cleaved PARP-1 in the Ramos cells treated during 8 h with the recombinant *Salmonella* strains (MOI of 100). Our finding shows the enhanced presence of cleaved PARP-1 in the cells treated with *S. enterica* L-SXTP as expected; these results are consistent with the high percentaje of Ramos cells with active caspase-3 and TUNEL positivity, described above. Since PARP-1 is a substrate of caspase-3 (32 KDa), we also analyzed this apoptotic molecule by Western blot. The presence of this former protein was detected in the cells treated with vincristine and also in the cells treated with the recombinant *Salmonella* strains; interestingly, the results showed a complete reduction in the expression of this molecule in the cells treated with *S. enterica* L-SXTP. This observation may be explained because the processing of this molecule toward active caspase-3 was observed in Figure 5A. These data suggest that the cell-permeable Bax BH3 peptide, expressed and released by *S. enterica* through the MisL autotransporter, antagonized the activity of the anti-apoptotic proteins in the Ramos cells, restoring the cellular death by apoptosis.

### Antitumor Activity of the Cell-Permeable Bax BH3 Peptide Expressed and Released From *S. enterica* in a Murine Xenograft Model of Human B NHL

**Figure 6:**
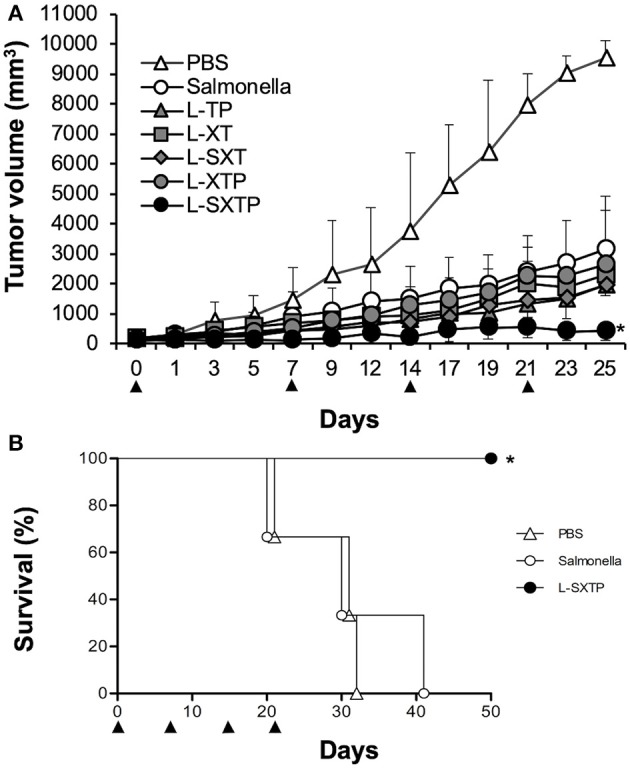
Antitumor activity of *S. enterica* L-STXP in a murine xenograft model of human B NHL. Athymic nu/nu BALB/c mice were subcutaneously injected with 10^7^ Ramos cells in the right flank. The treatment of the different groups was initiated when the tumors reached 100–150 mm^3^. The mice received 1 × 10^7^ CFU at days 0, 7, 14, and 21 (arrowhead), with each one of the recombinant *Salmonella* strains (L-TP, L-XT, L-SXT, L-XTP, and L-SXTP). Groups of mice treated with PBS 1× and non-transformed *S. enterica* SL3261 (*Salmonella*) were included as additional controls. **(A)** For the kinetics of tumor growth, the tumor size was analyzed daily with a Vernier caliper. In the graphics, the error bars represent the average ± *SD* of the tumor size (*n* = 5 mice/group). The results are representative of two independent experiments. ANOVA test was performed with Bonferroni *post hoc* for the difference between the groups. *p < 0.0001. **(B)** Survival curve after the tumor treatment. The data represent the percentage of survival (*n* = 3 mice/group). The results are representative of two independent experiments. *p = 0.02 by log-rank test.

**Figure 7:**
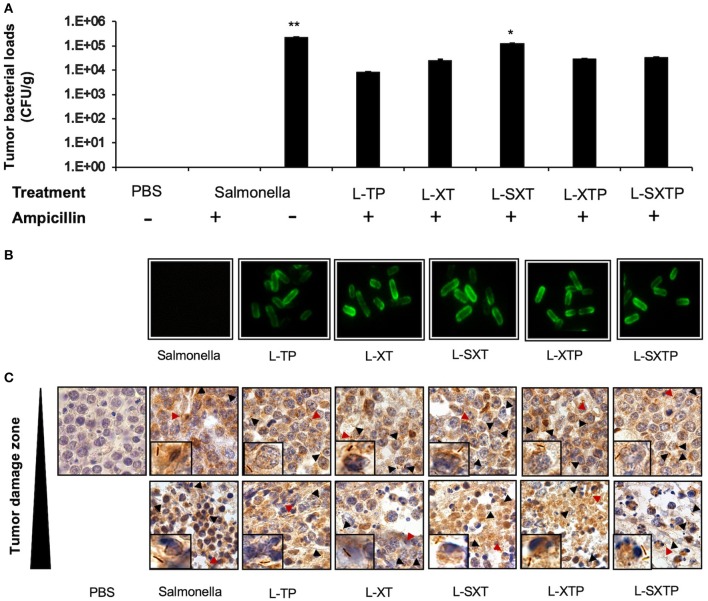
Tumor targeting of recombinant *Salmonella* strains in a murine xenograft model of human B NHL. Tumor-bearing mice were treated with four doses of 1 × 10^7^ colony-forming units (CFU) in the tail vein of the recombinant *Salmonella* strains and evaluated for the antitumor activity (tumor size) and euthanized after 5 days of the last inoculation. Tumors from those mice were resected and analyzed for tumor colonization. **(A)** Tumor suspensions were cultured in BHI agar medium in the presence or absence of ampicillin during 12 h at 37°C. The figure shows the number of colonies counted and reported as CFU per tissue gram (CFU/g). No *Salmonella* bacilli were observed in the group treated with PBS 1×. In the group of *S. enterica*, the bacteria grow only in the absence of ampicillin. **(B)** Subsequently, some recombinant bacteria isolated in agar BHI with ampicillin were analyzed for protein expression by immunofluorescence assay using an antibody anti-Flag-FITC. **(C)** The presence of the bacteria in the tumor tissue was also analyzed by performing an *in situ* immunohistochemical assay using an anti-*Salmonella* induced in rabbit as the primary antibody. The presence of *Salmonella* bacilli in brown color (arrowhead and close up) was detected in tumor sections with no histological changes and also in sections with necrosis/apoptosis areas (damaged zone). Images have 100× magnifications. The results are representative of three independent experiments. ANOVA test was performed with Bonferroni *post hoc* for the difference between the groups. For **(A)**, *p = 0.02, **p < 0.0001.

The antitumor effect of *S. enterica* L-SXTP, which expresses and releases from its surface the cell-permeable Bax BH3 peptide through the MisL autotransporter system, was analyzed in a murine xenograft model of human B NHL. Accordingly, athymic nu/nu female BALB/c mice from 6 to 8 weeks were implanted in the right flank with 10^7^ Ramos cells. Once the tumor reached 100–150 mm^3^ in size (approximately within 15 days after inoculation), groups of five mice were inoculated in the tail vein (1 × 10^7^ CFU) with each one of the previously induced recombinant *Salmonella* strains. Mice received four identical doses of bacteria with a 7-days interval. Control mice received PBS or non-transformed *S. enterica* SL3261 under the same inoculation scheme. The size tumor and survival were registered daily. Figure 6A shows that inoculation with recombinant *Salmonella* strains were performed at days 0, 7, 14, and 21. The analysis at day 25 (4 days after the fourth inoculation) shows a maximum reduction or the tumoral size in the mice that received *S. enterica* L-STXP (440 ± 330 mm^3^), this size corresponds to only 4% of the tumoral size development from the group that received PBS (9,527 ± 582 mm^3^, representing 100% of the size developed by the tumor). Decreased tumor volume, as compared with PBS-treated mice for the control groups were 33% with non-transformed *S. enterica* SL3261, 24% with *S. enterica* L-XT, 20% with *S. enterica* L-SXT, 20% with *S. enterica* L-TP, and 27% with *S. enterica* L-XTP. At day 25, the presence of the different recombinant *Salmonella* strains was also comfirmed in the tumors of the different treated groups; the recovered bacteria still showed the capacity to produce recombinant protein (Figure 7B). For the survival analysis, groups of three tumor-bearing mice were inoculated with PBS 1×, non-transformed *S. enterica* SL3261, and previously induced *S. enterica* L-STXP. For this assay, the groups were observed until 50 days. In Figure 6B, the extension of the survival is observed for up to 50 days from the mice that received the *S. enterica* L-STXP treatment compared with the group that received PBS 1× (the last mouse died around the 30th day) and the group that received non-transformed *S. enterica* SL3261 (the last mouse died around the 40th day). These findings altogether clearly prove the antitumor and therapeutic effect of the cell-permeable Bax BH3 peptide expressed and released from *S. enterica* through the MisL autotransporter system.

### Targeting of Recombinant *Salmonella* Strains to the Murine Xenograft Model of Human B NHL

The targeting of the different recombinant *Salmonella* strains to the murine xenograft model of human B NHL was analyzed by culture in BHI agar (in the presence or absence of ampicillin) of the homogenized xenograft tumor specimen previously treated with the recombinant *Salmonella* strains. Subsequently, the recombinant bacteria isolated in agar BHI with ampicillin were analyzed for protein expression by immunofluorescence assay. Figure 7A shows the presence of bacteria in all groups of mice inoculated with the recombinant *Salmonella* strains, and these bacteria still have the ability to express the recombinant proteins on its surface, as depicted in Figure 7B. To reinforce these findings, we also analyzed the presence of bacteria in tumor tissue. *In situ* immunohistochemical assay was performed using an antibody anti-*Salmonella* induced in rabbit, our results show the presence of 4–10 *Salmonella* bacilli per oil immersion field in all tumor-bearing mice treated with the recombinant *Salmonella* strains. No *Salmonella* bacilli were found in the group treated with PBS 1×. *Salmonella* bacilli were localized in tumor sections with no histological changes (undamaged) and also in sections with necrosis/apoptosis areas (damaged), as observed in Figure 7C. These results confirm that the recombinant *Salmonella* strains have targeted the tumor microenvironment of the murine xenograft model of human B NHL.

### The Cell-Permeable Bax BH3 Peptide Expressed and Released From *S. enterica* Induces Apoptosis in the Murine Xenograft Model of Human B NHL

**Figure 8:**
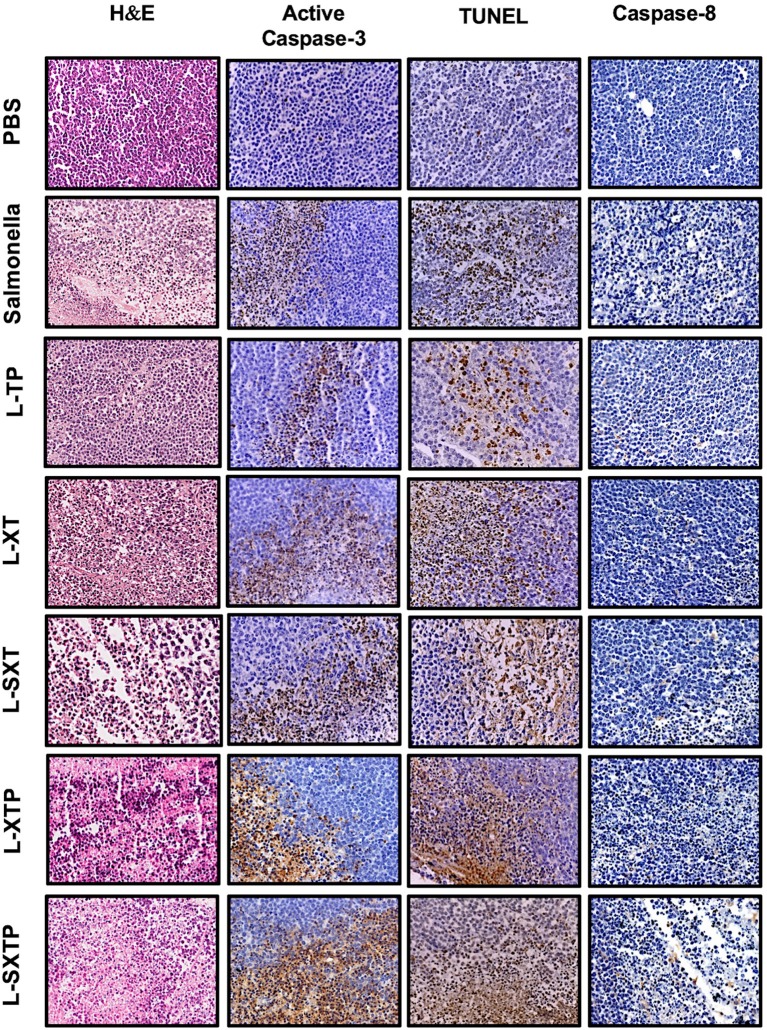
Apoptotic markers detected by immunohistochemistry in tumors treated with the recombinant *Salmonella* strains. Tumor-bearing mice were treated with four doses of 1 × 10^7^ colony-forming units (CFU) in the tail vein of the recombinant *Salmonella* strains and evaluated for the antitumor activity (tumor size) and were euthanized after 5 days of the last inoculation. Tumors from those mice were resected and analyzed for apoptotic markers as active caspase-3, TUNEL, and caspase-8. The column of hematoxylin and eosin staining shows a lymphoid neoplasm in all groups, composed of monomorphic medium-sized cells with a high mitotic rate. Macrophages and tumor-infiltrating lymphocytes were barely observed. Necrosis areas were detected in groups treated with the recombinant *Salmonellas* strains. For apoptotic markers, we used an antibody against active caspase-3 (1:250) and caspase 8 (1:1,500). The *in situ* TUNEL assay was performed following the instructions for the *in situ* Cell Death Detection Kit (Roche). In all cases, brown color represents positive cells. The columns of active caspase-3 and TUNEL shows a strong signal in the group treated with *S. enterica* L-SXTP. A small number of positive cells were observed for caspase-8 staining in all groups. Images have 20× magnification. The results are representative of three independent experiments.

The apoptosis induced by *S. enterica* L-SXTP was evaluated in the murine xenograft model of human B NHL that previously received four doses in the tail vein of the recombinant *Salmonella* strains and euthanized after 5 days of the last inoculation. Tumors from those mice were resected and prepared for histological analysis and immunohistochemical staining. Figure 8 shows representative images of staining tumors from each group. Hematoxylin and eosin staining shows a lymphoid neoplasm in all groups composed of monomorphic medium-sized cells with relatively uniform round to oval nuclei, multiple small nucleoli, and basophilic cytoplasm. These neoplasms also show a high mitotic rate and Ki67 proliferative index around 95% (data not shown). Macrophages and tumor-infiltrating lymphocytes were barely observed. Necrotic areas were detected in groups treated with the recombinant *Salmonellas* strains, but they were more evident in the group treated with *S. enterica* L-SXTP. Figure 8 also shows active caspase-3 and TUNEL-positive cells in all groups treated with the recombinant *Salmonellas* strains. However, treatment with *S. enterica* L-SXTP elicits the highest apoptosis, as expected. It is important to note that a strong signal of apoptotic markers was found in the necrotic areas. Caspase 8-positive cells were barely detected in the tumor-bearing mice with all treatments. These data confirm the activation of intrinsic apoptosis as the mechanism of antitumor activity mediated by the *S. enterica* L-SXTP in the murine xenograft model of human B NHL.

### Inflammatory Cytokines Are Elevated in the Murine Xenograft Model of Human B NHL Treated With *S. enterica* That Expresses and Releases the Cell-Permeable Bax BH3 Peptide

**Figure 9:**
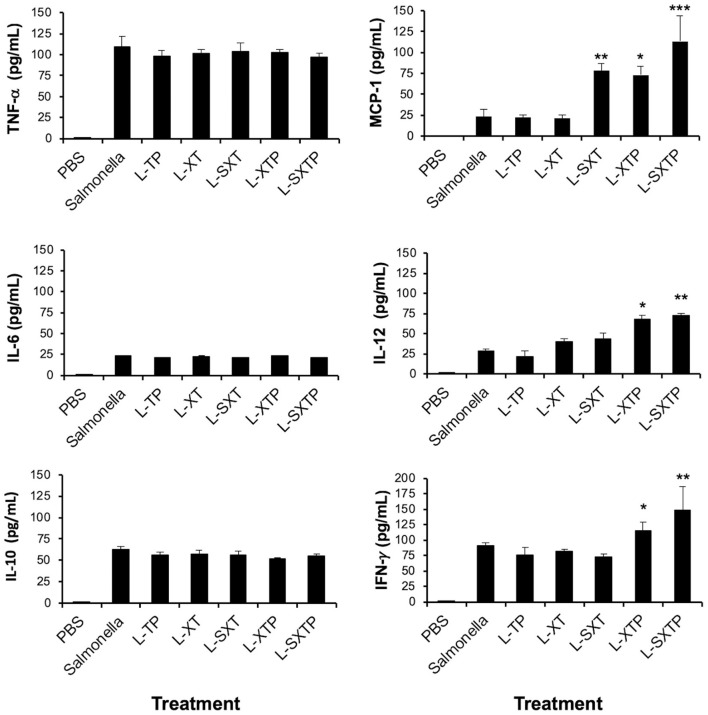
Inflammatory cytokines determined in serum from tumor-bearing mice treated with the recombinant *Salmonella* strains. Tumor-bearing mice were treated with four doses of 1 × 10^7^ colony-forming units (CFU) in the tail vein of the recombinant *Salmonella* strains and evaluated for the antitumor activity (tumor size) and were euthanized after 5 days of the last inoculation. Serum from those mice was analyzed for the presence of inflammatory cytokines as IL-6, IL-10, MCP-1, IFN-**γ**, TNF-α, and IL-12p70 using the Cytometric Bead Array (CBA) Mouse Inflammation Kit (BD Biosciences). Samples were acquired in the flow cytometry BD FACSCanto II, and the output was processed in the FCAP Array software v3.0. (BD Biosciences). The results are representative of three independent experiments. ANOVA test was performed with Bonferroni *post hoc* for the difference between the groups. For MCP-1, *p < 0.005, **p < 0.001, ***p < 0.0001; For IL-12, *p < 0.009, **p < 0.001; For IFN-γ, *p < 0.001, **p < 0.0002.

Given that the capacity of *S. enterica* L-SXTP to induce apoptosis in the murine xenograft model of human B NHL has been proven, we can wonder if the intravenous administration of these recombinant *Salmonella* strains has an effect on the inflammatory cytokine production. To approach this question, serum samples of the mice that had previously received four doses in the tail vein with the recombinant *Salmonella* strains and euthanized after 5 days of the last inoculation were analyzed by means of the CBA kit. This kit allows the detection of six inflammatory cytokines: IL-6, IL-10, MCP-1, IFN-**γ**, TNF-α, and IL-12p70. Figure 9 shows the comparison between the presence of every cytokine analyzed in the murine xenograft model, which had received the treatment with recombinant *Salmonellas* and the corresponding concentration values found for the control group of mice treated with PBS 1× (low average concentration values ranging from 0.34 to 0.42 pg/ml). The average concentrations of TNF-α observed for the different groups show values in the interval between 96.2 and 93.5 pg/ml, while between 20.4 and 23.1 pg/ml for IL-6, and between 51.1 and 62.3 pg/ml for IL-10. Unexpectedly, the group of mice treated with *S. enterica* L-SXTP showed high concentration values of cytokines as MCP-1 (113.1 pg/ml), IL-12 (72.1 pg/ml), and IFN-γ (148.2 pg/ml) compared with those values obtained from the group treated *S. enterica*: MCP-1 (23.1 pg/ml), IL-12 (27.7 pg/ml), and IFN-γ (89.8 pg/ml). These findings clearly suggest that *S. enterica* L-SXTP stimulates the production of inflammatory cytokines with antitumoral activity, in addition to inducing the expression and the release of the cell-permeable Bax BH3 peptide.

## Discussion

In the last years, the survival of patients with NHL has increased substantially (4, 5), nevertheless, the development of drug resistance limits the complete success of the treatments (6) and sets the guidelines for the research and development of new antitumor therapies that can completely eradicate drug-resistant transformed cells (7). The drug resistance has been associated mostly to the aberrant inhibition of the signals of intrinsic apoptosis, in which the genes and proteins of the Bcl-2 family play a very important role (8, 9). In the cells, the balance between survival or death is controlled by the members of the three groups of this family of proteins: the group of multidomain anti-apoptotic proteins (Bcl-2, Bcl-_XL_, Bcl-w, Mcl-1, and A1) promotes the survival of the cells by inhibiting the pro-apoptotic proteins; the group of multidomain pro-apoptotic proteins (BH1-3) (Bax, Bak, and Bok), which are apoptosis effectors; and the group of pro-apoptotic BH3-only proteins (Bid, Bim, Puma, Noxa, Bad, Bmf, Hrk, and Bik), which are the apoptosis initiators (17–19). In the healthy cells, the anti-apoptotic proteins attach and inhibit the effector proteins Bax or Bak, blocking their polymerization on the mitochondrial surface and avoiding the apoptosis initiation (20). The single BH3-only proteins are induced in response to stress signals and promote the apoptosis binding directly to the effector proteins or to the anti-apoptotic proteins to release the effector proteins (27). According to this balance, the overexpression of this anti-apoptotic proteins in the tumor cells favors the survival of the transformed cell and represents a treatment resistance mechanism (61).

In NHL, it has been documented that the overexpression of anti-apoptotic proteins Bcl-2 (13, 14), Bcl-_XL_ (15, 16), and Mcl-1 (62) is associated to drug-resistant profile. Reverting this resistance mechanism has been possible due to the structural studies that reveal that proteins from the Bcl-2 family interact with each other through a hydrophobic groove formed by their BH domains (17–19), and that peptides derived from the BH3 domain of pro-apoptotic proteins can bind to the anti-apoptotic proteins, antagonizing their function (22–24). *In vitro* assays using hydrophobic peptides from the BH3 domain of the proteins Bax, Bad, and Bak, when binding them to the fusogenic peptide of the antennapedia protein to make them permeable to the tumor cells of head and neck squamous cell carcinoma, antagonized the activity of Bcl-_XL_ and Bcl-2 and restored the apoptosis (25). The concept of eliminating the tumor cells blocking the activity of the anti-apoptotic proteins has also been successful using the small molecules that mimic the function of the BH3-only proteins, as ABT-737(63) and its oral bioavailable derivative ABT-263/Navitoclax) (64), GX15-070/Obatoclax (65), and ABT-199/Venotoclax; this last one was recently approved by the FDA for the treatment of CLL (26, 27) but not for NHL.

Despite its effectiveness and promising results, the peptides from the BH3 domain and the mimetic molecules from BH3 domain still need to be specifically and selectively directed toward the tumor microenvironment in order to decrease side effects. In order to solve this problem, in this work, we propose to use a live attenuated bacterium to carry the peptide from the BH3 domain of the Bax protein directly into the tumor microenvironment and allow the access to the cytosol of the NHL tumor cells. *S. enterica* serovar Typhimurium, gram-negative and anaerobic facultative bacteria, is probably the most studied live attenuated bacterium in the therapy against cancer due to its high affinity for tumor tissue (28, 29), favored by hypoxia, acidity, necrosis, and release of metabolites that can act as chemoattractant for this bacterium in solid and semisolids tumors, its great intrinsic antitumor potential (66, 67), its ability to activate the innate and adaptive antitumor immune response (30), and its great capacity of use as delivery system because once it is in the tumor microenvironment, it turns into a true factory of heterologous molecules (31, 32), as cytokines (68–71), chemokines (72), ligands of death (73, 74), pro-apoptotic proteins domains (75), and peptides (76), among others (30). Furthermore, safe strains are available such as *S. enterica* serovar Typhimurium VNP20009, mutant in *msbB* and *pur I* genes, with a reduced toxicity associated with lipopolysaccharide, and this bacterial strain was demonstrated to be safe and tolerable in clinical trials (77). Recently, it has been reported that *S. enterica* SL3261 (Aro A, mutant in the aromatic amino acids) was used to express anti CD20 antibodies and drug converting enzymes to eradicate human lymphomas (78), and our group has shown the success of *S. enterica* SL3261 to carry and transfer plasmids into tumor cells. Transferred plasmid that encodes a peptide from the BH3 domain of the pro-apoptotic Bax protein antagonized the anti-apoptotic activity of the Bcl-2 family proteins, restored the apoptosis, and induced chemosensitization in tumor cells (33).

In the present study, we evaluated the feasibility of the cell-permeable Bax BH3 peptide (constituted by the Bax-BH3 peptide, bound to the molecular Tag Flag and to the fusogenic peptide) expressed and released from the surface of *S. enterica* SL3261 through the MisL autotransporter system (L-SXTP) to promote apoptosis signaling and death of NHL cells. Previously, we have reported that the MisL autotransporter system can be used to express heterologous molecules on the surface of *S. enterica* (35), and these molecules were released to the microenvironment by a cleavage site of OmpT protease (34, 58).

In this work, using molecular modeling of the L-SXTP complex, we showed for the first time an approximation of the 3-D structure of the MisL autotransporter and each one of the components of the cell-permeable Bax BH3 peptide. This 3-D structure allowed us to confirm that MisL is an autotransporter protein (79) constituted by a β barrel domain of 12 antiparallel chains with five short handles that internalize toward the periplasmic space and one extracellular α domain that presents toward the edge N-terminal a combination of several units of β-strand secondary structure, organized in a specific geometry of β-solenoid architecture, the cell-permeable Bax BH3 peptide consolidates an α-helix folding and the cutting site for the OmpT protease, revealing itself as a flexible loop; these do not induce an unfolding or instability of the 3-D structure of the MisL autotransporter neither in its global configuration nor in the vicinity to the β-solenoid (Figures 1A,B). For the theoretical prediction of the internalization of the cell-permeable Bax BH3 peptide in the membrane, we observed that due to its amino acid composition and its hydrophobicity, it is possible that it crosses the membrane through a diffusion process (Figure 1C). With this information, and using molecular biology, we obtained the *S. enterica* L-STXP, which expresses and releases from its surface the cell-permeable Bax BH3 peptide through the MisL autotransporter, and we also obtained the controls: *S. enterica* L-XT (control that expresses the Bax-BH3 peptide), *S. enterica* L-SXT (control that expresses and releases the Bax-BH3 peptide), *S. enterica* L-TP (control that expresses the fusogenic peptide), *S. enterica* L-XTP (control that expresses the Bax-BH3 peptide and the fusogenic peptide and keeps it anchored to the bacteria membrane), all the recombinant proteins expressed the molecular Tag flag (T) and the MisL autotransporter (L) (Figures 2A,B). The introduction of the plasmids and the induction of the protein did not affect the growth of *S. enterica* (data not shown). As expected, our results showed that the peptides of interest were expressed and translocated to the surface of *S. enterica* through the MisL autotransporter, as shown in the Western blot assays, immunofluorescence, and flow cytometry in Figure 3; the immunoelectron microscopy also shows that the recombinant protein L-SXTP was expressed on the surface of the *S. enterica* L-SXTP (Supplementary Figure 1). These data confirm the functionality of the MisL autotransporter in its ability to translocate proteins to the surface of *S. enterica* (34, 35, 58) and are consistent with the observations of the molecular modeling in which it described that the addition of the cell-permeable Bax BH3 peptide to the α domain of the MisL autotransporter does not affect its structure.

We further evaluated the effect of the recombinant *Salmonella* strains over the viability of Ramos cells that come from a Burkitt lymphoma, an aggressive human B NHL that expresses Bcl-_XL_ and Mcl-l (Figure 4A). With this aim, Ramos cells were treated at different times with the recombinant *Salmonella* strains to a MOI of 100. As depicted in Figure 4B, the differential effect can be observed gradually between the 8 and 10 h. In this assay, the recombinant strain of *S. enterica* L-SXTP induced the death of 39% of the NHL cells at 8 h, with an increase to almost double at 10 h (69%). Interestingly, the *Salmonella* strain that expresses the cell-permeable Bax BH3 peptide on its surface but does not release it (*S. enterica* L-XTP) induced an even lesser rate of death (16% at 8 h and 27% at 10 h) to the observed values for the non-transformed bacteria *S. enterica* SL3261 (21% at 8 h and 36% at 10 h). This observation suggests that it is necessary to release the cell-permeable Bax BH3 peptide from the bacteria surface for induction of cell death. The other recombinant *Salmonella* strains (L-XT, L-SXT, and L-TP) showed lesser values compared to the ones induced by non-transformed *S. enterica* SL3261. These data were confirmed in the viability assays 8 and 10 h after the treatment of Ramos cells with the different recombinant *Salmonella* strains (Figures 4C,D). A slight increase of the cell death observed with non-transformed *S. enterica* SL3261 can be mediated by the intrinsic oncolytic activity of the bacteria (30) due to the release of the nitrate reductase that metabolizes nitrates and nitrites to nitric oxide, a molecule that has the ability to induce cellular apoptosis (80), and by the induction of autophagy due to the increase in proteins such as Beclin-1 and LC3 (81).

The apoptosis assays performed to Ramos cells treated with the different recombinant *Salmonella* strains during 8 h confirm that the cellular death induced by *S. enterica* L-SXTP in the viability assays is due to the restoration of the apoptosis mechanism. As expected, the Ramos cells that received the treatment with *S. enterica* L-SXTP showed twice of active caspase-3 cells and TUNEL positivity than the Ramos cells that received the treatment with the non-transformed *S. enterica* SL3261 and other controls (Figures 5A,B). These results were consistent with the increase of cleavage PARP-1 (substrate of caspase-3) in the group treated with *S. enterica* L-SXTP compared with the controls (Figure 5C). In this group, complete caspase-3 was not evident (Figure 5C), however, and increasing amount of cleavage form of caspase-3, detected as active caspase-3-positive cells, was observed (Figure 3A). Interestingly, the high rate of active caspase-3-positive cells observed in the group with vincristine does not induce highest rate of TUNEL-positive cells as expected, these data were consistent with low expression of cleaved PARP-1. The slight increase in the apoptosis, mediated by non-transformed *S. enterica* SL3261, can be due to the intrinsic oncolytic activity of *S*. *enterica* aforementioned. These results are consistent with previous reports in which the use of Bax-BH3 peptides bound to an antennapedia fusogenic peptide restored the apoptosis in head and neck squamous cell carcinoma and acute leukemia cells (25).

To demonstrate the antitumor effect of the recombinant *Salmonella* strains, we developed a murine xenograft model of human B NHL using Ramos cells, which were implanted in the right flank of athymic nu/nu female BALB/c mice, and after 15 days, when the tumors reached between 100 and 150 mm^3^, mice were inoculated in the tail vein (1 × 10^7^ CFU) with each one of the previously induced recombinant *Salmonella* strains. Mice received four identical doses of bacteria with a 7-days interval. Our results showed that the mice that received the treatment with *S. enterica* L-SXTP significantly reduced the tumor size during the 26 days after the first treatment. Interestingly, the groups of mice inoculated with recombinant *Salmonella* strains as control (L-XT, L-SXT, L-TP, and L-XTP) also showed tumor reduction, at intermediate sizes, compared with the group that only received treatment with PBS 1*. This effect could be due to the intrinsic oncolytic activity of *S. enterica* and its ability to activate the innate immunity (30), which is still present in this athymic mice, and can be mediated by the presence of dendritic cells, neutrophils, macrophages, and natural killer tumor-infiltrating cells (66, 82). In the tumor-bearing mice treated with the recombinant *Salmonella* strains, the presence of *Salmonella* bacilli that still produce recombinant proteins at day 26 (Figures 7A,B) confirms the *S. enterica* ability to reach the tumor microenvironment, and once there, express the cell-permeable Bax BH3 peptide through the MisL autotransporter system. This observation may be promoted by the capacity of *S. enterica* to infect B lymphocytes (83) and was consistent with studies that claim that these bacteria colonize solid and semisolid tumors (32). In addition, the group that received *S. enterica* L-SXTP also increased the survival in the 50 days that the study lasted (Figure 6B) compared with the group that received PBS (31 days) and the group that was treated with non-transformed *S. enterica* SL3261 (41 days). In this last group, the intrinsic oncolytic activity mechanisms and the activation of the innate immune response mediated by *S. enterica* (30) were sufficient to improve 10 more days the mice survival compared with those that received PBS. These findings clearly prove the antitumor and therapeutic effect of the cell-permeable Bax BH3 peptide expressed and released from *S. enterica* through the MisL autotransporter system. The histological analysis of the tumor-bearing mice that received four doses of recombinant *Salmonella* strains and euthanized after 5 days of the last inoculation (day 26) shows a lymphoid neoplasm composed of monomorphic medium sized cells, with scarce macrophages and tumor-infiltrating lymphocytes, with the presence of necrotic areas mostly in the group treated with *S. enterica* L-SXTP. The presence of recombinant bacteria observed in tumor sections with or without histological changes (Figure 7C) confirm the targeting of the *S. enterica* strains to the murine xenograft model of human B NHL and is consistent with the tumor targeting of *Salmonella* reported in other murine models of lymphoma (53, 84). The presence of apoptotic marker as active caspase-3 and TUNEL-positive cells was detected in all groups treated with the recombinant *Salmonella* strains; however, the effect was more evident in the tumor treated with *S. enterica* L-SXTP. These *in situ* results (Figure 8) are consistent with the *in vitro* assays, where the enhanced active caspase-3-positive cells correlate with higher TUNEL-positive cells and enhanced cleaved PARP-1 (Figure 5). The presence of apoptotic markers in the groups treated with the non-transformed *S. enterica* and others controls can be due to the intrinsic oncolytic activity of *S. enterica* aforementioned (30). It is important to mention that caspase-8 was almost negative in all groups (Figure 8), suggesting that the extrinsic apoptosis does not play any role in the antitumor activity mediated by the recombinant *Salmonella* strains used as treatment.

Since the histology analysis barely identified some immune cells as macrophages and tumor infiltrating lymphocytes in the murine xenograft model of human B NHL treated with the recombinant *Salmonella* strains, we further analyzed the systemic production of inflammatory cytokines in the serum of the tumor-bearing mice treated with the recombinant *Salmonella* strains. Inflammatory cytokines IL-6, TNF-α, MCP-1, IFN-**γ**, and IL-12p70 that have been associated with antitumor activity were identified (53, 84–86), and also an anti-inflammatory cytokine was detected (IL-10) (85) (Figure 9). Surprisingly, the tumor-bearing mice inoculated with *S. enterica* L-SXTP shows more than two-fold concentrations of the MCP-1, IFN-**γ**, and IL-12p70 cytokines compared with the group that received non-transformed *S. enterica* SL3261 and controls. These findings suggest that the antitumor and therapeutic effect of the cell-permeable Bax BH3 peptide expressed and released from *S. enterica* through the MisL autotransporter system may be enhanced with the presence of inflammatory cytokines with antitumor activity, an event that requires further investigation.

Taken together, our findings represent an important step forward in demonstrating the potential of live attenuated *S. enterica* serovar Typhimurium strain SL3261 expressing and releasing cell-permeable Bax-BH3 peptide through the MisL autotransporter system as an eventual alternative to treat relapsed or refractory NHL.

## Data Availability Statement

All datasets generated for this study are included in the article/Supplementary Material.

## Ethics Statement

All protocols for animal experimentation were carried out in accordance with procedures authorized by the Children's Hospital Federico Gomez Ethical committee for Animal Experimentation, Mexico City, to whom this project was previously submitted.

## Author Contributions

RL-P conceived, supervised the project, designed the experiments, analyzed the data, and wrote the manuscript. AM-C and PM-L generated the recombinant proteins, performed the viability, and apoptosis assays. EB-B, LF-M, and GB-G participated in the in vivo experiments. DP-G and LM-V performed the molecular modeling of the MisL autotransporter system. UJ-H performed the bacteria tumor targeting experiments. BC-M generated the immunoelectron microscopy data. LC-M participated in the histology and immunohistochemical analysis.

### Conflict of Interest

The authors declare that the research was conducted in the absence of any commercial or financial relationships that could be construed as a potential conflict of interest.
