# Supplementary material for: Live Attenuated Salmonella enterica Expressing and Releasing Cell-Permeable Bax BH3 Peptide Through the MisL Autotransporter System Elicits Antitumor Activity in a Murine Xenograft Model of Human B Non-hodgkin's Lymphoma
Source: Front Immunol. 2019 Nov 14;10:2562. doi: 10.3389/fimmu.2019.02562 (PMC6874163; doi:10.3389/fimmu.2019.02562)
Supplement: Supplementary file 1 [file Data_Sheet_1.pdf]

Supplementary Figures

Material and Methods.

Suplementary Figure 1.

Cell-permeable Bax BH3 Peptide expressed on the surface of the *Salmonella enterica* through the MisL Autotranporter: Detection by Dot Blot, Flow cytometry, and ImmunoElectron Microscopy.

To reinforce the evidence about the expression of Cell-permeable Bax BH3 Peptide expressed on the surface of *Salmonella enterica* through the MisL Autotransporter, bacterium strains *Salmonella enterica*, *Salmonella enterica* SXTP (containing the plasmid that codifies for the Cell-permeable Bax BH3 Peptide, with no MisL autotransporter, expressed in bacteria cytoplasm), and *Salmonella enterica* L-SXTP (with the plasmid codifying for the Cell-permeable Bax BH3 Peptide, expressed on the surface through MisL autotransporter), were treated for induction in thioglycollate broth supplemented with DHB and ampicillin (Sigma-Aldrich) to promote anaerobic environment, and incubated for 12 hours, at 37 ºC and shaken at 200 rpm as described previously. After that, the protein expression was evaluated by Dot Blot, Flow Cytometry, and Immunoelectron Microscopy.

a) DotBlot. Bacterial pellets (10^9 CFU) were resuspended in 500 µL of PBS 1X, and sonicated for 6 minutes on ice with 30 sec pulse on, 15 sec pulse off (30% Vibration amplitude) in the sonicator (130 Watts, Vibra-Cell, SONICS), protein quantification was performed with the Bicinchoninic Acid kit by ThermoFisher Scientific. For the Dot Blot, 100 µg of total protein was dripping onto nitrocellulose membrane, and dry for one hour. After that, the membrane was blocked one hour with 5% dry milk in PBST (0.05% of Tween 20 in PBS 1X) and washed with PBST. The Immunodetection of the recombinant proteins was performed with a monoclonal antibody anti-Flag induced in mouse (1mg/mL, Sigma-Aldrich), diluted 1:1000 in PBA 1X (1% BSA in PBS 1X). The membranes were incubated for 2 hours with the primary antibody and subsequently for 1 hour with the anti-mouse IgG-HRP secondary antibody (1mg/mL, Abcam) at room temperature, diluted 1:1000 in PBA 1X. Finally, the membranes were developed in a solution of 4-chloro-α-naphtol (Sigma-Aldrich) in Methanol-PBS 1X, pH 7.4 and Hydrogen Peroxide (J.T. Baker).

b) Flow cytometry. 10^8 bacteria were incubated with the antibody anti-Flag FITC induced in mouse (1 mg/mL, Sigma-Aldrich) at a dilution of 1:100 in PBA 1X for 2 hours and 100 rpm at room temperature in dark conditions. The bacterial suspension was washed in PBS 1X and resuspended in 450 µL PBS 1X for Flow Cytometry Analysis in the CytoFLEX system (Beckman Coulter).

c) Immunoelectron microscopy. Bacterial pellets (10^9 CFU) were fixed with 0.5% glutaraldehyde and 4% paraformaldehyde in PBS 1X for 1 hour at room temperature, washed with PBS 1X and dehydrated with increasing concentrations of ethanol before embedded in LR White resin (London Resin Co), that was polymerized at 60 °C overnight. Thin sections (60 nm) were obtained and recovered in nickel grids. Sections were incubated overnight at 4 °C with mouse anti-Flag (not diluted, Sigma-Aldrich) as the primary antibody, washed and incubated by 1 hour with the gold-conjugated anti-mouse IgG (1:40 dilution) as the secondary antibody (Ted Pella Inc) (with 20 nm gold particles). As a negative control, the secondary antibody alone was used. Samples were washed and contrasted with uranyl acetate and lead citrate for their analysis in a JEM-1011 transmission electron microscope (JEOL).

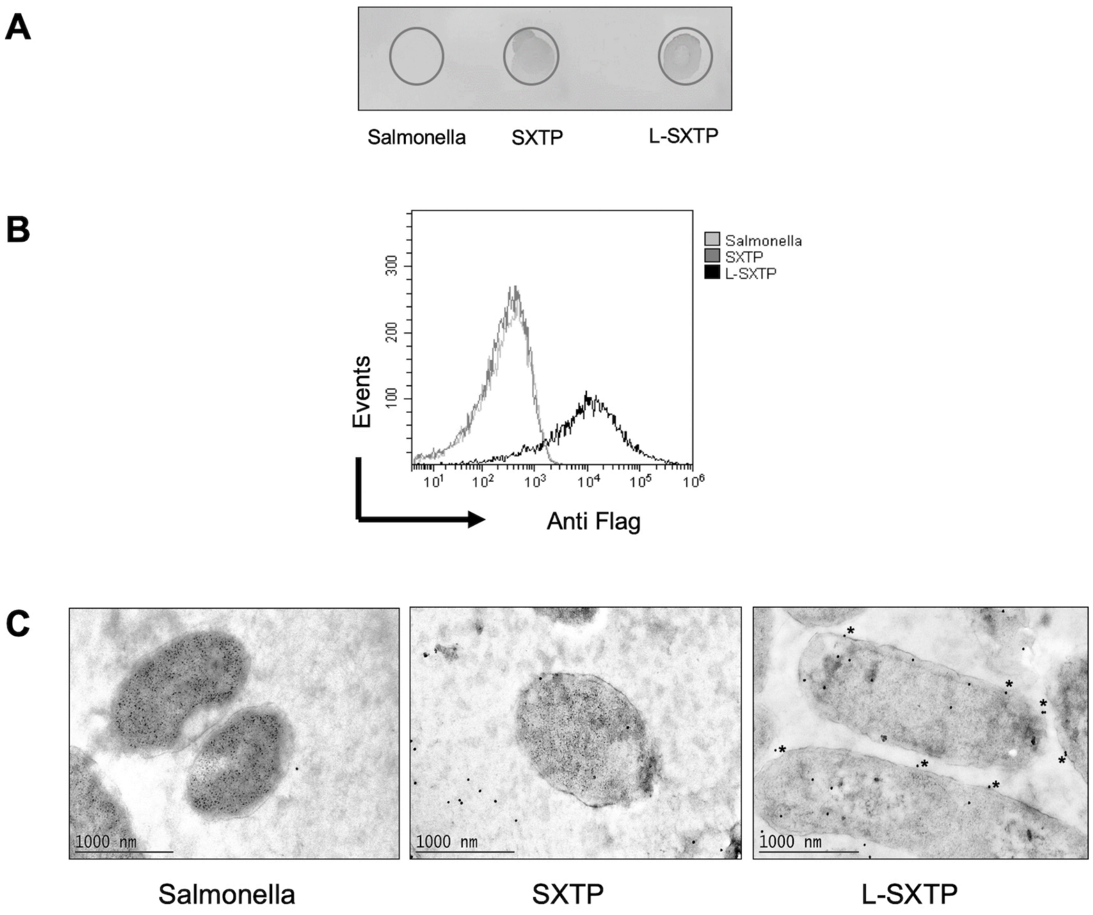

Supplementary Figure 1. Cell-permeable Bax BH3 Peptide is expressed on the surface of the *Salmonella enterica* through the MisL Autotransporter System. The expression and translocation of the Cell-permeable Bax BH3 Peptide on the surface of *Salmonella enterica* through the MisL autotransporter system were evaluated after 12 hours of induction in a thioglycollate medium. The evaluation was performed with *Salmonella enterica* SL3261(Salmonella), *Salmonella enterica* SXTP (SXTP) and *Salmonella enterica* L-SXTP (L-SXTP) strains. (A) The Dot Blot was performed with total bacterial extracts and incubated with a mouse anti-Flag antibody followed by an anti-mouse IgG-HRP. Salmonella was used as the negative control, and the positive spot was detected in SXTP and L-SXTP strains. (B) The Flow cytometry was performed with bacterial suspensions of Salmonella, SXTP, and L-SXTP. The immunostaining was performed in non-permeabilized conditions with the antibody anti-Flag-FITC induced in mice. Controls in the flow cytometry (histogram to the left) are Salmonella and SXTP strains (express the Cell-permeable Bax BH3 Peptide in the bacteria cytosol). Positive staining (histogram to the right) was observed with L-SXTP strain (express the Cell-permeable Bax BH3 Peptide, on the bacterial membrane through the MisL autotransporter system). (C) Immunoelectron microscopy was performed with bacterial suspension. Bacteria were immunolabelled with a mouse anti-Flag antibody, followed by a secondary antibody anti-mouse IgG that has been coupled to 20 nm gold particles. Gold particles were detected on the outer membrane of L-SXTP strain and in the cytosol of both SXTP and L-SXTP strains. No signal was detected in the Salmonella strain. The asterisk shows the protein on the bacterial membrane. Results are representative of three independent experiments

Suplementary Figure 2.

Detection of apoptosis by Terminal deoxynucleotidyl transferase dUTP Nick End Labeling (TUNEL) assay.

After the infection of Ramos cells with recombinant Salmonella strains, 50,000 cells were immobilized on silanized slides using cytospin and fixed with acetone. The DNA fragmentation in tumor cell lines was evaluated by TUNEL assay using an *In Situ* Cell Death Detection Kit (HRP) (Roche Applied Science, Mannheim, Germany) following the manufacturer's instructions. An Antigen Unmasking Solution Citrate-Based (Vector), incubated in a water bath for 20 minutes was used for antigen retrieval, and endogenous peroxide activity was blocked with methanol and 3% hydrogen peroxide for 15 minutes. Then they were blocked with normal horse serum for 1 hour and the preparations were incubated with TUNEL reaction mixture (ROCHE) for 1 hour. Subsequently incubated with an anti-fluorescein antibody (Converter-POD) for 30 min, at 37 °C in a humidified atmosphere in the dark. The color was generated by adding the substrate 3,3'-diaminobenzidine (DAB) (Vector) for 1 to 2 minutes, and counterstaining was performed with hematoxylin. Subsequently, the cells were dehydrated and covered with resin. Finally, the slides were analyzed under light microscopy (Olympus BX-40) and counted the percentage of nuclear positive cells.

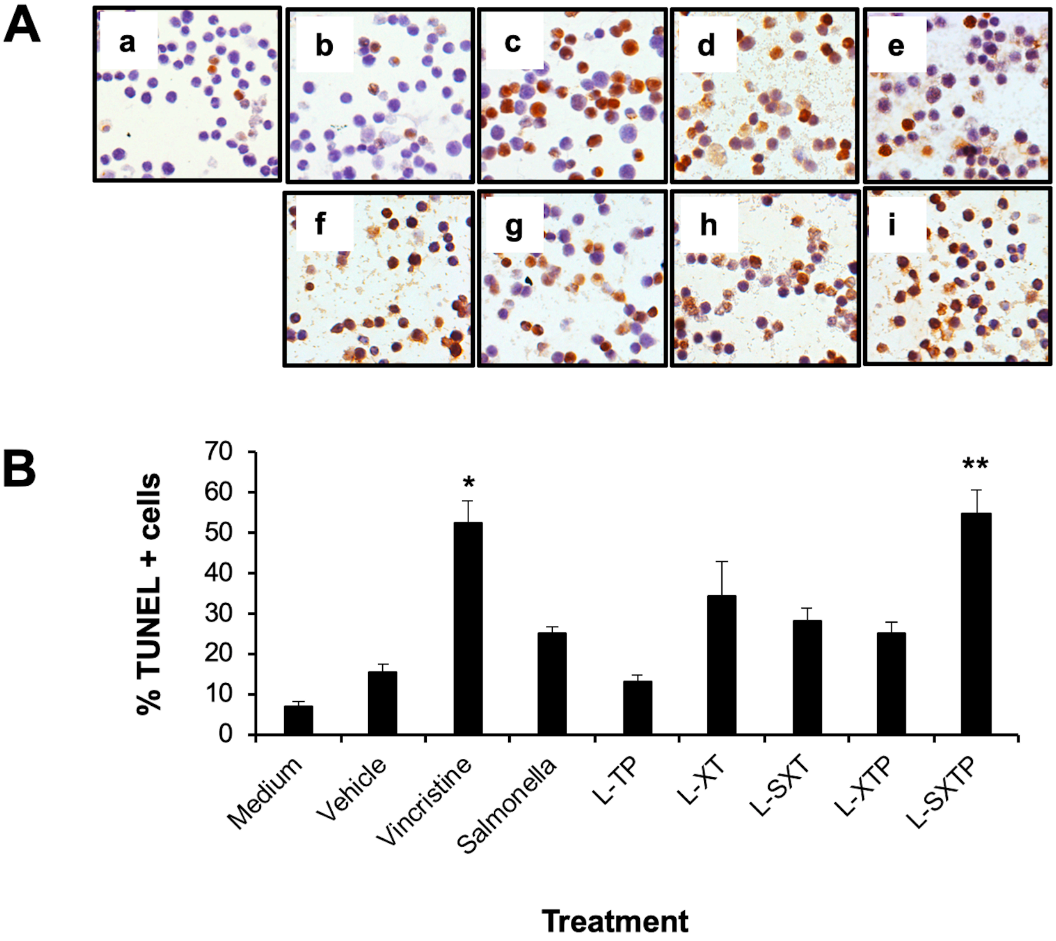

Supplementary Figure 2. ***Salmonella enterica*** **L-STXP induces apoptosis of Ramos cells.** Ramos cells were infected during 8 hours to a MOI of 100 with the different recombinant Salmonella strains (L-TP, L-XT, L-SXT, L-XTP y L-SXTP). As controls we used: non-treated cells (Medium) as a negative control, sterile water as a solvent to Vincristine (Vehicle), non-transformed *Salmonella enterica* SL3261 (Salmonella) and vincristine 0.5 nM as a positive control. The apoptosis was analyzed by determination of TUNEL positive cells. (A) Representative micrographs show the TUNEL positive cells: a) Medium, b) Vehicle, c) Vincristine, d) Salmonella, e) L-TP, f) L-XT, g) L-SXT, h) L-XTP, and i) L-SXTP. Magnification 40X. (B) Quantification of TUNEL positive cells. In the plot, error bars represent the average DS of 3 independent experiments. ANOVA test was performed with Bonferroni Post Hoc for the difference between the groups. *, ***$p<0.0001$*.
